# Two-directional synthesis as a tool for diversity-oriented synthesis: Synthesis of alkaloid scaffolds

**DOI:** 10.3762/bjoc.8.95

**Published:** 2012-06-06

**Authors:** Kieron M G O’Connell, Monica Díaz-Gavilán, Warren R J D Galloway, David R Spring

**Affiliations:** 1 Department of Chemistry, University of Cambridge, Lensfield Rd, Cambridge, CB2 1EW, UK

**Keywords:** alkaloids, cascade reactions, chemical diversity, diversity-oriented synthesis, Lewis acid catalysis, two-directional synthesis

## Abstract

Two-directional synthesis represents an ideal strategy for the rapid elaboration of simple starting materials and their subsequent transformation into complex molecular architectures. As such, it is becoming recognised as an enabling technology for diversity-oriented synthesis. Herein, we provide a thorough account of our work combining two-directional synthesis with diversity-oriented synthesis, with particular reference to the synthesis of polycyclic alkaloid scaffolds.

## Introduction

Diversity-oriented synthesis (DOS) aims to prepare structurally diverse compound collections in an efficient manner [1–3]. Of the possible “types of diversity” that can be incorporated into a compound collection, the most important, in terms of creating a functionally (biologically) diverse collection, is generally considered to be *scaffold* (or *skeletal*) *diversity*, i.e., the variation of molecular frameworks between compounds [4–5]. Therefore, one of the key challenges in DOS is the development of strategies that allow the efficient generation of a range of complex molecular scaffolds. A large number of approaches towards this goal have been reported, with some of the most effective being based around the “folding-up” of functionalised linear substrates into cyclic molecular architectures [6–8]. The design and synthesis of these linear substrates can, in itself, represent a significant challenge as it is desirable that these compounds are easily accessible in a small number of synthetic steps. Two-directional synthesis [9–12] offers a powerful method for the synthesis of such substrates, because each synthetic transformation has the potential to provide twice as much molecular complexity compared to standard approaches.

We have recently reported a strategy for DOS that combined two-directional synthesis with the use of these folding reaction pathways [13]. In this work, two-directional synthesis was used to rapidly generate a series of linear aminoalkenes, which were then folded into bicyclic and tricyclic scaffolds through Lewis acid mediated cascade processes. The compounds produced in this campaign were reminiscent of naturally occurring alkaloids, such as the *Coccinellidae* natural products, which are secreted by ladybirds to deter predators [14]. The total synthesis of one of their number, myrrhine, was also achieved by the elaboration of one of the compounds produced. In this article, the work is presented in more detail, alongside additional results from our work combining two-directional synthesis with DOS. Treated together, we believe these works provide useful insights into the potential utility of two-directional synthesis as an enabling technology for DOS.

**Figure 1:**
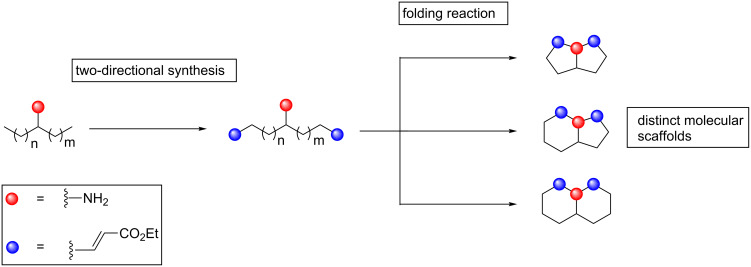
Overview of the DOS strategy.

The initial DOS campaign was largely inspired by the pioneering work of Robert Stockman on combining two-directional synthesis with tandem reactions to create complex molecular architectures [15–18]. The key folding step in the DOS was the Lewis acid mediated pairing reaction of a nucleophilic amino group with suitable electrophilic functionality, provided by Michael acceptor α,β-unsaturated ester groups. Two-directional synthesis was used to append these electrophilic groups at two positions around the linear substrates, allowing bicyclisation processes to be instigated. The scaffold diversity between the products then resulted from the different ring sizes that it was possible to form from these linear substrates. (Figure 1 shows an overview of the DOS strategy.)

## Results and Discussion

### Synthesis of linear precursors

**Scheme 1:**
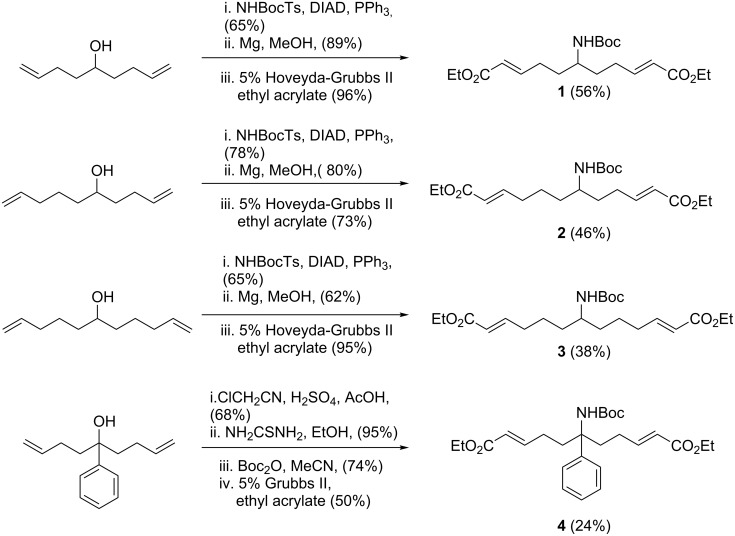
Synthesis of linear cyclisation precursors **1**–**4**.

Our initial studies explored the synthesis and reactivity of four *N*-Boc-aminoalkenes containing α,β-unsaturated ester groups (**1**–**4**, Scheme 1), as substrates for intramolecular pairing reactions. The *N*-Boc protecting group was chosen to give the potential for deprotection to be carried out in tandem with the Lewis acid catalysed cyclisation reactions by intramolecular conjugate addition. Three of these compounds (**1**–**3**) were obtained from the corresponding alcohols [19–20] in three steps: Mitsunobu reaction with NH-Boc-tosylate, followed by tosyl deprotection with magnesium, and finally two-directional cross metathesis with ethyl acrylate to install the desired α,β-unsaturated ester functionality. This sequence provided the desired *N*-Boc-aminoalkenes in respectable overall yields of 38–56%. Compound **4** was prepared in a four-step sequence from the requisite phenyldialkyl alcohol. Ritter reaction with chloroacetonitrile followed by cleavage of the resulting chloroacetamide with thiourea gave the free amine [21], which was then protected with Boc anhydride. Finally, cross metathesis with ethyl acrylate furnished the desired compound **4** in 24% overall yield.

### Cyclisation reactions

**Scheme 2:**
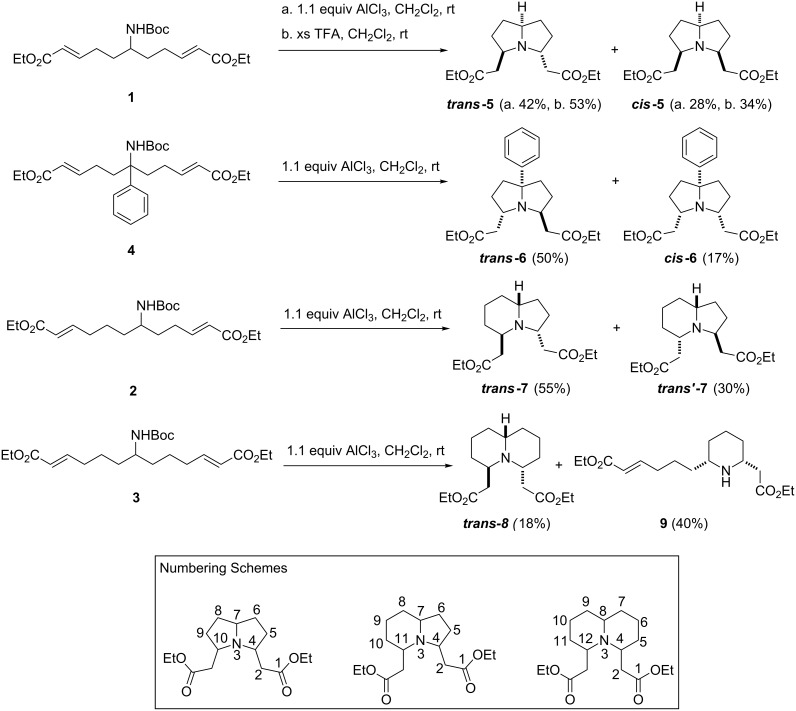
AlCl_3_ catalysed tandem Boc-removal/bicyclisation processes; the yields quoted refer to the isolated yields of single compounds.

The first attempts at the tandem Boc-deprotection/bicyclisation of these substrates were performed by using AlCl_3_ as the Lewis acid (Scheme 2); compounds **1**–**4** were treated with 1.1 equiv of AlCl_3_ in dichloromethane at room temperature. These conditions proved effective at promoting bicyclisation for compounds **1**, **2** and **4**, for which the desired bicyclic products were obtained in 67–85% yield, as a mixture of diastereomers. The cyclisation of **1** gave pyrrolizidine **5** as a mixture of 4,10-*trans*-7,10-*trans (****trans-*5***)* (42%) and 4,10-*cis*-7,10-*trans (****cis-*5***)* (28%) isomers, which proved to be separable by flash chromatography. The stereochemistry of ***cis*-5** was confirmed by analogy to known ^1^H and ^13^C NMR values [22] and by NOESY spectroscopy, which showed enhancements between H-7, H-4 and H-10. In this case, it proved possible to achieve an improved yield of both diastereomers by treating **1** with an excess (163 equiv) of trifluroacetic acid, which furnished ***trans*-5** and ***cis*-5** in 53% and 34% yield, respectively. The corresponding reaction of phenyl-substituted analogue **4** also gave a mixture of two diastereomers; the major diastereomer was 4,10-*trans*-7,10-*cis* isomer (***trans*-6**), which was formed in 50% yield, and a 17% yield of the 4,7,10-*cis-*isomer (**cis-6**) was also obtained*.*

Indolizidine **7** was produced in very good overall yield (85%). The unsymmetrical nature of this compound gave rise to the possibility of formation of additional diastereomers when compared to **5** and **6**; however, once again only two were formed in any appreciable amount. The compounds obtained were both found to have a *trans*-fused geometry at the ring junction, as indicated by IR (strong absorbance at 2850 cm^−1^) and ^1^H NMR (H-7 chemical shift around 2.4 ppm) spectroscopy [23–24]. The ester-bearing side chains were also found to be *trans* to each other in both cases, meaning that the two products obtained differ from each other only in which ring has the side chain *cis* to the ring junction hydrogen. The favoured product was the 4,11-*trans*-7,11-*cis* isomer (***trans-*7**) in which the side chain of the 6-membered ring is *cis* to the ring junction proton; this compound was isolated in 55% yield. The alternative 4,11-*trans*-4,7-*cis* isomer (***trans’*-7**) was obtained in 30% yield. Despite the good yields obtained for these three examples, the cyclisation of **3** under these conditions proved disappointing, with 4,12-trans-8,12-cis-quinolizidine (***trans*-8**) only obtained in 18% yield, along with 40% of monocyclic species **9**.

**Scheme 3:**
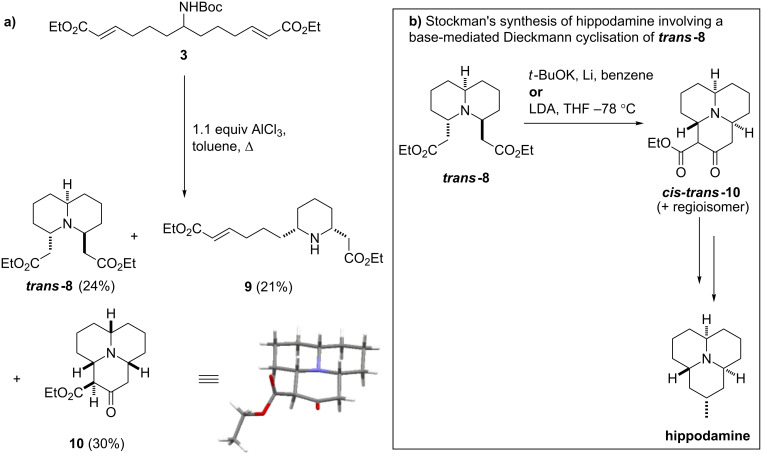
(a) AlCl_3_ catalysed formation of tricyclic alkaloid **10** along with an X-ray crystal structure of **10**; (b) base-mediated Dieckmann cyclisation of ***trans*-8** employed by Stockman and co-workers in the synthesis of hippodamine [25].

In light of the difficulties encountered in producing the desired bicyclic species, an optimisation study of the cyclisation of **3** was undertaken, which resulted in a number of interesting findings that are summarised in Table 1. The initial alterations made little difference to the process; increasing the reaction time up to seven days had essentially no effect on the product ratio, with ***trans*-8** and **9** obtained in 21% and 37% yield, respectively, and increasing the temperature to the point of reflux in dichloromethane also had little effect on the conversion. However, when the reaction solvent was changed to toluene and the reaction mixture heated to reflux, ***trans*-8** and **9** were still formed in similar proportions (24% and 21%), but in this case an additional product was also isolated in 30% yield. This compound was found to be tricyclic compound **10** (Scheme 3a), which was obtained as a single diastereomer with all of the ring junction protons on the same face. This all–*cis-*stereochemistry was surprising, as so far **8** had only been obtained with the side chains in *trans*- configuration; however the configuration of **10** was unambiguously confirmed by X-ray crystallography. In some ways the formation of a tricyclic species such as **10** was not altogether surprising, as a similar tricyclic species was generated in Stockman’s synthesis of hippodamine [25]. In that work, bicycle ***trans*-8** was transformed into the corresponding tricyclic compound (possessing *cis–trans* ring junction stereochemistry) by a base-mediated Dieckmann cyclisation (Scheme 3b). It seems in our case that, under the correct conditions, a Dieckmann reaction can be made to occur in tandem with the Boc-deprotection and double-conjugate addition processes. This one-pot, four-step reaction process is extremely interesting for the amount of molecular complexity generated in a single transformation, and also because the all-*cis*-stereochemistry of **10**, which differs from the *cis–trans*-stereochemistry observed by Stockman for the Dieckman cyclisation of ***trans*-8**. For these reasons, further investigations into the process were carried out.

**Table 1:** Overview of the Lewis acid mediated folding reactions of **3**.

solvent	Lewis acid (equiv)	temp.	% yield			
			***trans*-8**	***cis*-8**	**9**	**10**

DCM^a^	AlCl_3_ (1.1)	rt	18	—	40	—
DCM^b^	AlCl_3_ (1.1)	rt	21	—	37	—
toluene^a^	AlCl_3_ (1.1)	reflux	24	—	21	30
toluene^a^	AlCl_3_ (3)	reflux	43	—	13	—
toluene^a^	Sc(OTf)_3_ (3)	reflux	50	—	21	—
toluene^a^	Sn(OTf)_2_ (0.5)	reflux	—	9	—	72
toluene^a^	Sn(OTf)_2_ (1.1)	reflux	30	13	—	23
toluene	Sn(OTf)_2_ (3)	reflux	29	—	10	—

^a^Reaction stirred overnight; ^b^reaction stirred for seven days.

The amount of AlCl_3_ was increased to 3 equiv; however, this led to the suppression of tricycle formation in favour of a slight increase in the yield of ***trans*-8** to 43% (a 13% yield of **9** was also obtained). Switching the Lewis acid to Sc(OTf)_3_ and still using 3 equiv, gave a slight improvement in the yields of the ***trans*-8** to 50% and **9** to 21% but no formation of **10**. Switching the Lewis acid again to Sn(OTf)_2_ resulted in a decrease in the yields of ***trans*-8** to 29% and **9** to 10% and again no formation of **10**. The amount of Lewis acid was then reduced to 0.5 equiv, which dramatically altered the course of the reaction. Performing the reaction under reflux in toluene with 0.5 equiv Sn(OTf)_2_ produced tricycle **10** in 72% isolated yield. Thus both ***trans*-8** and **10** could be accessed in good yields from the same substrate simply by varying the amount and identity of the Lewis acid used.

Interestingly, the catalytic variant of the reaction also produced 9% of the bicyclic ***cis*-8**, which had not been isolated from any of the previous reactions. For completeness, one further reaction was performed by using 1.1 equiv of Sn(OTf)_2_; careful purification of this reaction gave 23% of **10**, 30% of ***trans*-8** and 13% of ***cis*-8**. The presence of the previously undetected ***cis*-8** in these two final reactions was intriguing and led us to speculate as to whether a different mechanistic pathway could be in operation depending on the amount of Lewis acid used.

**Scheme 4:**
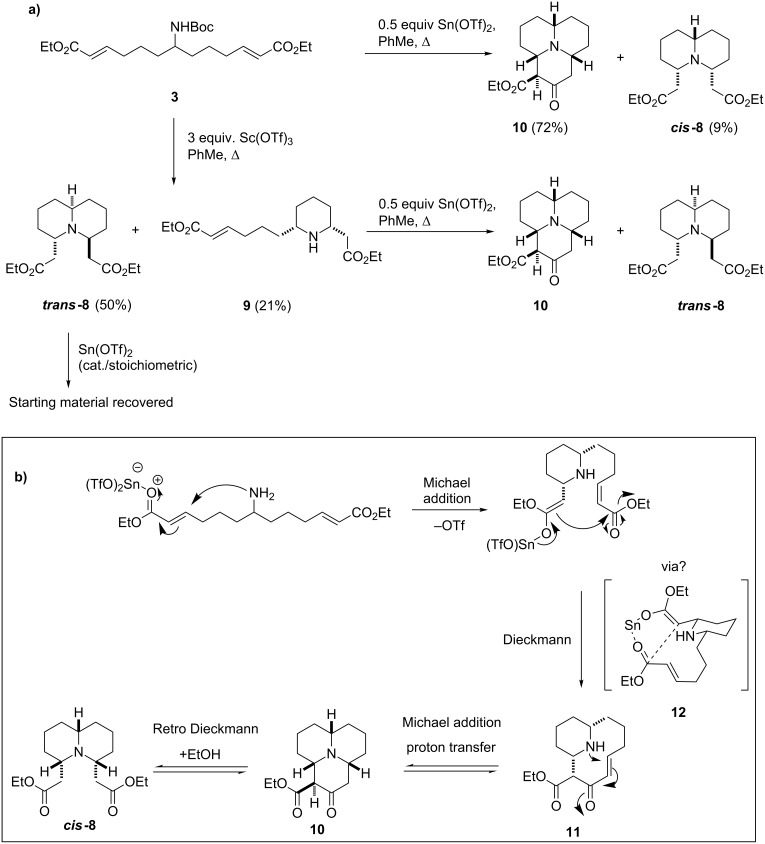
**(**a) Optimal conditions to obtain ***trans*-8** and **10** and the control experiments carried out to probe the mechanism of the process; (b) proposed mechanistic pathway for the tricyclisation of **3**.

A number of factors led to this mechanistic speculation; principal among them was the fact that ***cis*-8** was never detected in reactions in which **10** was not formed. In all of the earlier experiments the only bicycle detected was ***trans*-8**, implying that the double-conjugate addition process heavily favours the formation of this compound. Therefore it was considered that ***cis*-8** could be forming from **10**; suggesting, somewhat counter intuitively, that the Dieckmann cyclisation to give 6,10-bridged bicycle **11** could in some cases be favoured over the expected double-conjugate addition. Transannular conjugate addition across the 10-membered ring of **11** would give **10**, and a retro-Dieckmann process could then form ***cis-8***. Several control experiments were run in an attempt to validate this hypothesis (Scheme 4a).

***Trans*-8** was treated with both stoichiometric and catalytic amounts of Sn(OTf)_2_ and there was no evidence of tricycle formation in either case, with only starting material recovered from the reactions. The direct formation of **10** from ***trans*-8** was not thought to be possible due to their differing stereochemistry; however, an alternative diastereomer of **10** may be expected to form (as observed by Stockman for the corresponding base-mediated process) [25]. Monocycle **9** was treated with catalytic Sn(OTf)_2_, which led to a mixture of ***trans*-8** and **10**. As the formation of **10** from ***trans***-**8** had been proven not to occur, it seemed reasonable to assume from this that it is possible to form both species from **9**. It is also noteworthy that the best yields of **10** were obtained when the reaction was performed while fitted with a Dean–Stark apparatus containing pieces of sodium to trap the ethanol formed in the Dieckmann cyclisation, and thus inhibit the retro-Dieckmann reaction. Finally, **10** was treated with catalytic Sn(OTf)_2_, which, as expected, gave ***cis*-8**, suggesting that the retro-Dieckmann reaction does occur.

Taking these results into account, we tentatively suggest that the mechanism for the tricyclisation of **3** does indeed proceed via a Dieckmann cyclisation to form the 10-membered ring followed by transannular conjugate addition (Scheme 4b). The fact that this appears to occur when lower amounts of Lewis acid are used (0.5 or 1.1 equiv) but not when three equiv are used indicates that the mechanistic path of the cascade is dependent on the amount of the Lewis acid present. When three equiv of Lewis acid are present (the “stoichiometric” process), it is feasible that during the course of the reaction both of the carbonyl groups are activated simultaneously, allowing the double-conjugate addition to proceed smoothly. However, when fewer equiv are used (the “catalytic” process) there is a relative deficiency of Lewis acid present, and thus, it is less probable that both carbonyl groups can be coordinated to separate Lewis acid molecules simultaneously. If the Dieckmann reaction occurs via a standard 6-membered transition state, it requires both carbonyls to be either coordinated to, or bonded to, a single metal centre, and so this could go some way towards explaining the apparent course of the reaction. Assuming that the Lewis acid remains bonded to the carbonyl of the enol form of the ester group after the first conjugate addition, there may be insufficient Lewis acid free in solution to activate the second ester group separately, leaving it effectively inert to conjugate addition (and Dieckmann cyclisation). However, if the second carbonyl group becomes coordinated to the metal centre that is bonded to the first, the Dieckmann reaction becomes the most favourable process and so occurs preferentially to the second conjugate addition. For these reasons, we cautiously postulate that the Dieckmann reaction may occur via a chelated transition state such as **12**, in which both carbonyls are coordinated to a single metal centre.

The data in Table 1 provides further support for the suggestion that different mechanisms can operate depending on the amount of Lewis acid used. This support is provided by the fact that the formation of **10** proceeds best under truly catalytic conditions: when 0.5 equiv Sn(OTf)_2_ were used, a 72% yield of **10** was obtained compared to 23% when 1.1 equiv were used. In fact, it appears that when 1.1 equiv of Lewis acid are used both mechanisms can occur, as indicated by the formation of both ***trans*-8** and **10** in these reactions, but when 0.5 equiv are used the formation of ***trans*-8** is not observed suggesting that under these conditions the simple double-conjugate addition cannot occur. The identity of the Lewis acid used in the reaction seems not to overtly affect the course of the reaction in terms of the products obtained, as similar product distributions were observed for the reactions using three equiv of AlCl_3_, Sc(OTf)_3_ and Sn(OTf)_2_. The formation of **10** was also not limited to the use of Sn(OTf)_2_, as a 30% yield of **10** was obtained for the reaction using 1.1 equiv of AlCl_3_.

**Scheme 5:**
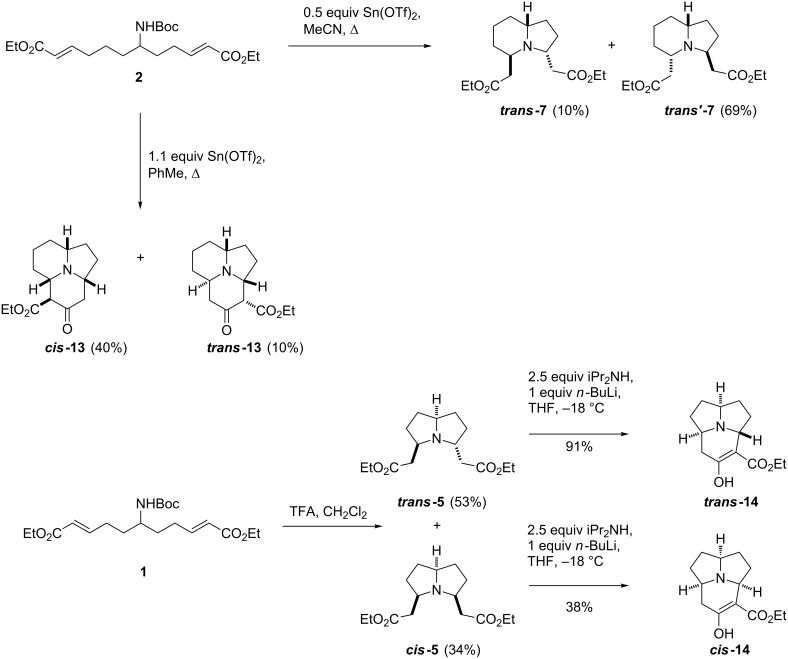
DOS of 5-5-6 and 5-6-6 tricyclic alkaloids **13** and **14**.

Between them, the catalytic and stoichiometric variants of this reaction provide a useful illustration of the use of reagent-based diversification within a predominantly substrate-based strategy. Attempts were then made to apply this reagent-based diversification to the other linear substrates (Scheme 5). Compound **2** was treated with 0.5 equiv of Sn(OTf)_2_ in acetonitrile, which surprisingly did not lead to the formation of the 6-6-5-tricyclic species, instead producing ***trans*’-7** in 69% yield along with 10% ***trans*-7**. While this reaction did not produce any tricyclic species, it was an interesting result, as the selectivity for ***trans*’-7** over ***trans*-7** was the opposite of that observed for the original AlCl_3_ catalysed process. Performing the reaction in toluene with 1.1 equiv of Sn(OTf)_2_ did produce the expected tricyclic species in 50% yield as a mixture of two diastereomers (***cis*-13** and ***trans*-13**) in a 4:1 ratio, proving that the folding of this linear substrate into tricyclic species in one-pot was also possible. Unfortunately, this did not prove to be the case for **1**; all attempts to transform **1** into a tricyclic species in one pot, using either stoichiometric or catalytic Lewis acid were unsuccessful. However, it did prove possible to perform the Dieckmann reaction on the bicyclic compounds ***cis*-5** and ***trans*-5** to access the tricyclic architectures. The bicyclic species were treated with LDA at −18 °C, which affected the desired cyclisation in both cases. Strangely, the process proved far more efficient for ***trans*-5,** giving ***trans*-14** in 91% yield, compared to 38% for the *cis*-isomer. In both cases the ^1^H NMR suggested that these compounds exist as the usually disfavoured enol tautomer.

These reactions, along with the reactions of **1**, clearly illustrate the power of this two-directional approach to DOS. Using this methodology it was possible to access five bicyclic and tricyclic scaffolds covering a range of 3D shapes, including pyrrolizidines, indolizidines and quinolizidines, along with 6-6-6 and 5-6-6 azatricyclic species in a single transformation from a small collection of structurally simple linear starting materials. One further tricyclic scaffold (5-5-6) was also accessible in one further synthetic step. The effective introduction of reagent-based diversification into the strategy was extremely satisfying, as by altering the choice of Lewis acid and reaction temperature we were able to adjust the course and selectivity of the reactions to generate different scaffolds and stereochemistry. This combination of reagent and substrate-based approaches for the generation of molecular diversity can afford many interesting possibilities not achievable by either approach alone.

### Total synthesis of myrrhine

**Scheme 6:**
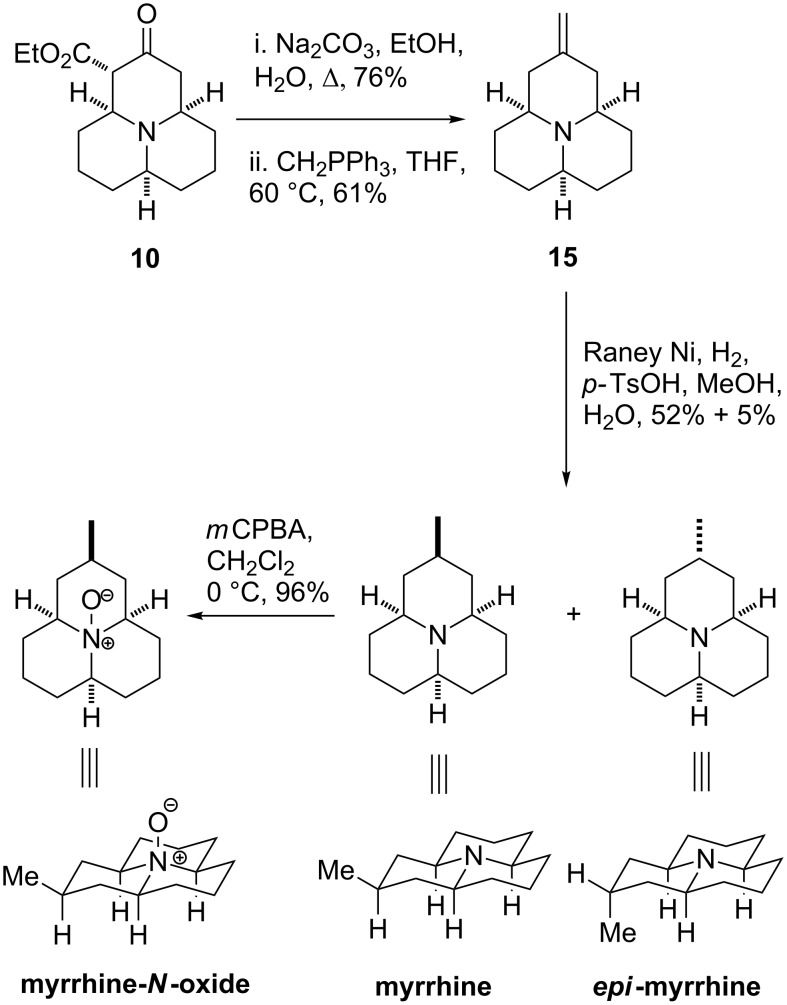
Total synthesis of myrrhine, *epi*-myrrhine and myrrhine-*N*-oxide.

Inspired by Stockman’s syntheses of the related alkaloids hippodamine and *epi*-hippodamine [26], tricyclic species **10** was identified as a potential intermediate for the total synthesis of myrrhine, which was then achieved in three steps from **10** (Scheme 6). Compound **10** was treated with Na_2_CO_3_ in a mixture of EtOH and H_2_O under reflux to achieve ester saponification, which was followed by decarboxylation, proceeding smoothly to give the corresponding ketone in 76% yield. The ketone was then transformed to the exocyclic alkene **15** in 61% yield by Wittig reaction with the appropriate phosphonium salt. The final step in the synthesis was a diastereoselective reduction of the double bond with hydrogen gas and Raney-nickel. This was achieved in a moderate 57% yield and with good (~10:1) diastereoselectivity by complexing the nitrogen lone pair with tosic acid, effectively blocking the undesirable face of the tricycle during the course of the reduction. This reduction also produced 5% of the unnatural (or as yet undiscovered) isomer *epi*-myrrhine. The *N*-oxide of myrrhine, which has also not been isolated from natural sources, was then synthesised in 96% yield by treating myrrhine with *m*CPBA. This synthesis of myrrhine compares favourably with previously reported syntheses [26–28], achieving the feat in eight steps and 7% overall yield.

### Alternative starting materials

The evident efficiency of two-directional synthesis in a DOS context, as exemplified by our synthesis of these alkaloid scaffolds, has led us to continue investigations in this area and to explore the potential utility of this approach for a range of different substrates. Among these substrates, two that stand out as particularly promising are nitromethane and tris(hydroxymethyl)aminomethane (Tris) **16**.

Nitromethane is of great interest to us as a potential DOS substrate, as we have a long-standing interest in developing divergent reaction pathways from small and simple starting materials [29–30]. As it represents a one-carbon unit, nitromethane is an ideal substrate for investigation. For Tris, the central quarternary carbon centre is the key point of interest, as we believe that, with the judicious selection of appendages, it should allow us to access a range of bicyclic structures, including examples of fused, bridged and spiro bicycles. Preliminary studies into the utility of these substrates in a DOS context have yielded some promising results.

**Scheme 7:**
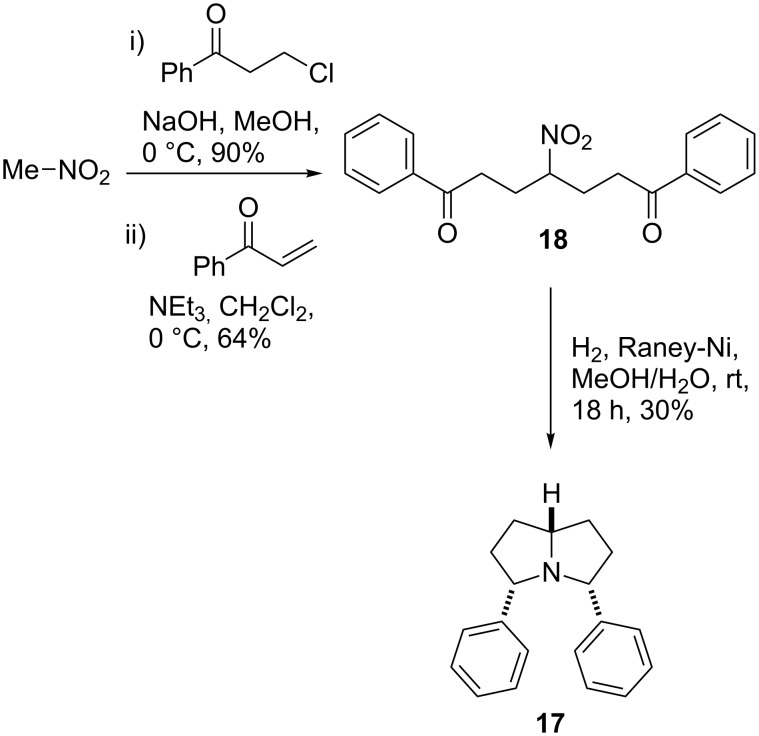
Use of nitromethane in DOS: Synthesis of *meso*-diphenylpyrrolidizine **17**.

Our work with nitromethane has so far led to the synthesis of *meso-*diphenylpyrrolizidine **17**, which was achieved in three steps (Scheme 7). Nitromethane was treated with NaOH, and the resulting anion was used to displace the chloride from 3-chloro-1-phenylpropan-1-one giving a 90% yield of the nitroketone. Two-directional synthesis of diketone **18** in this fashion did not prove to be feasible; however, it was achieved in good yield by Michael addition of the nitroketone anion to phenylvinylketone. Subjecting diketone **18** to H_2_ gas and Raney-nickel then reduced the nitro group and effected the desired double reductive amination to give **17** in 30% yield. It is likely that the scope of this sequence could be extended to include indolizidine and quinolizidine scaffolds, and so provide an alternative route to these frameworks, instead of the double Michael addition strategy.

**Scheme 8:**
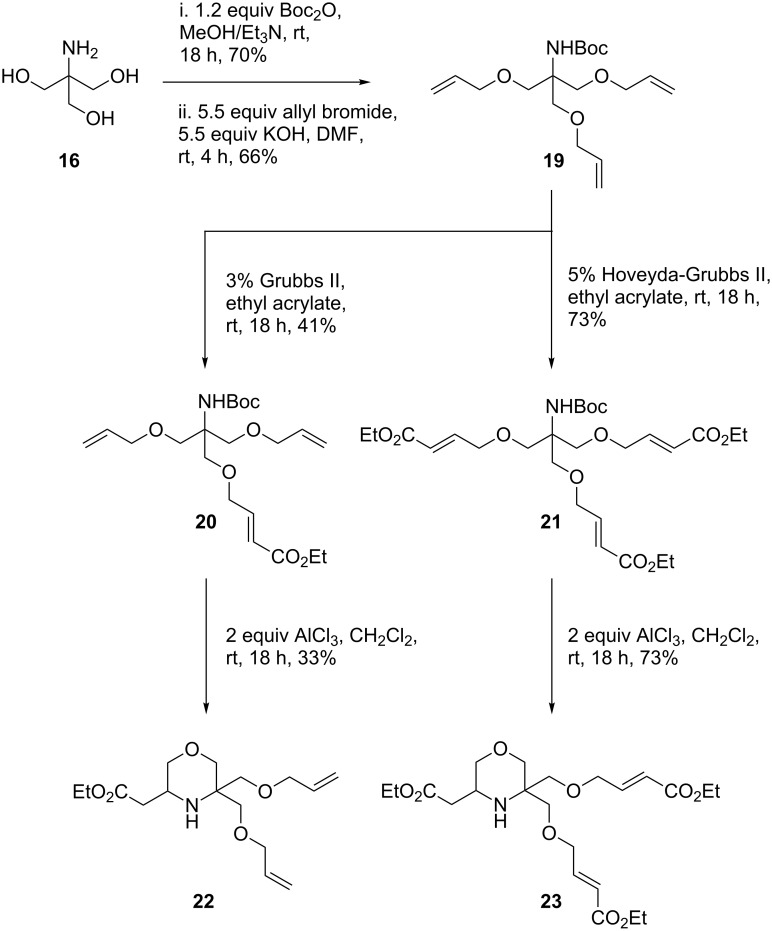
Use of Tris as a substrate for DOS: Synthesis of decorated morpholines **22** and **23**.

The three hydroxy and single amino groups of Tris give the potential for many variations in substitution; however, our studies have so far focused on allylated derivatives, in particular the triallyl derivative **19** (Scheme 8). The synthesis of **19** was achieved in two steps from Tris: *N*-Boc protection proceeded in 70% yield, and was followed by alkylation with an excess of allyl bromide to provide the desired triallyl species in 66% yield. Cross metathesis of **19** with ethyl acrylate was then performed. Fortunately, it proved to be possible to achieve some selectivity for different products by varying the catalyst used. Treating **19** with 3% Grubbs II catalyst in neat ethyl acrylate at room temperature gave monoester **20** in 41% yield, whereas performing the reaction with 5% Hoveyda-Grubbs II catalyst gave a 73% yield of triester **21**.

These two metathesis products were then subjected to the tandem deprotection-cyclisation conditions, this time by using two equiv of AlCl_3_ in dichloromethane at room temperature. For monoester **20**, only a single conjugate addition was possible, leaving the pendant terminal alkene groups as handles for further reactivity. This cyclisation proceeded to give the expected, functionalised morpholine scaffold **22** in a moderate 33% yield. A similar product was also obtained for **21**, with a single conjugate addition process occurring to give **23** in 73% yield and no trace of the bicyclic product detected. A number of attempts were then made to force the reaction to occur, by varying the Lewis acid to Sn(OTf)_2_ and Sc(OTf)_3_ and by heating under reflux in toluene, all of which produced varying amounts of **23**, with no trace of the bicyclic product detected. This result was disappointing and also, to some degree, surprising given the relative ease of formation of the corresponding quinolizidine compounds. We speculate that the relative difficulty in the reaction is due to the reduced availability of the lone pair of the morpholine nitrogen compared to the piperidine nitrogen (p*K*_a_ 8.36 versus 11.22) [31], retarding the rate of nucleophilic attack. Another possibility is that the oxygens in the pendant chains can form a stable hydrogen-bonded species with the NH group that inhibits the reaction of the nitrogen with the Michael acceptor ester groups. Studies involving the use of Tris as a potential DOS substrate remain on-going within our laboratories.

## Conclusion

The work presented in this article serves to illustrate the potential power of two-directional synthesis in DOS. The use of two-directional synthesis allowed us to access a range of bicyclic and tricyclic molecular scaffolds, rapidly and efficiently, by following a common reaction scheme. The nature of the two-directional synthesis lends itself to the formation of bicyclic compounds by the folding up of doubly substituted precursors, and it proved to be a very effective strategy for the synthesis of natural-product-like alkaloid scaffolds. Our work so far in this area has focused mainly on the synthesis of fused bicyclic compounds; however, we hope in the future to be able to apply a two-directional synthesis approach to the DOS of a wide range of molecular scaffolds and structural classes.

## Supporting Information

File 1 Experimental procedures and spectral data for all previously unreported compounds.

File 2 NMR Spectra of novel compounds.

## References

[1] Galloway W R J D, Isidro-Llobet A, Spring D R (2010). Nat Commun.

[2] Nielsen T E, Schreiber S L (2008). Angew Chem, Int Ed.

[3] Spandl R J, Díaz-Gavilán M, O’Connell K M G, Thomas G L, Spring D R (2008). Chem Rec.

[4] Galloway W R J D, Díaz-Gavilán M, Isidro-Llobet A, Spring D R (2009). Angew Chem, Int Ed.

[5] Dow M, Fisher M, James T, Marchetti F, Nelson A (2012). Org Biomol Chem.

[6] Burke M D, Berger E M, Schreiber S L (2003). Science.

[7] Morton D, Leach S, Cordier C, Warriner S, Nelson A (2009). Angew Chem, Int Ed.

[8] O’Leary-Steele C, Pedersen P J, James T, Lanyon-Hogg T, Leach S, Hayes J, Nelson A (2010). Chem–Eur J.

[9] Poss C S, Schreiber S L (1994). Acc Chem Res.

[10] Vrettou M, Gray A A, Brewer A R E, Barrett A G M (2007). Tetrahedron.

[11] Kennedy A, Nelson A, Perry A (2005). Beilstein J Org Chem.

[12] Gignoux C, Newton A F, Barthelme A, Lewis W, Alcaraz M-L, Stockman R A (2012). Org Biomol Chem.

[13] Díaz-Gavilán M, Galloway W R J D, O'Connell K M G, Hodkingson J T, Spring D R (2010). Chem Commun.

[14] Happ G M, Eisner T (1961). Science.

[15] Stockman R A (2000). Tetrahedron Lett.

[16] Stockman R A, Sinclair A, Arini L G, Szeto P, Hughes D L (2004). J Org Chem.

[17] Rejzek M, Stockman R A, van Maarseveen J H, Hughes D L (2005). Chem Commun.

[18] Robbins D, Newton A F, Gignoux C, Legeay J-C, Sinclair A, Rejzek M, Laxon C A, Yalamanchili S K, Lewis W, O’Connell M A (2011). Chem Sci.

[19] Boyer F-D, Hanna I (2006). Eur J Org Chem.

[20] Schwartz B D, McErlean C S P, Fletcher M T, Mazomenos B E, Konstantopoulou M A, Kitching W, De Voss J J (2005). Org Lett.

[21] Jirgensons A, Kauss V, Kalvinsh I, Gold M R (2000). Synthesis.

[22] Scarpi D, Occhiato E G, Guarna A (1999). J Org Chem.

[23] Crabb T A, Newton R F, Jackson D (1971). Chem Rev.

[24] Rader C P, Young R L, Aaron H S (1965). J Org Chem.

[25] Rejzek M, Stockman R A, Hughes D L (2005). Org Biomol Chem.

[26] Ayer W A, Dawe R, Eisner R A, Furuichi K (1976). Can J Chem.

[27] Mueller R H, Thompson M E, DiPardo R M (1984). J Org Chem.

[28] Gerasyuto A I, Hsung R P (2007). J Org Chem.

[29] Wyatt E E, Fergus S, Galloway W R J D, Bender A, Fox D J, Plowright A T, Jessiman A S, Spring D R (2006). Chem Commun.

[30] Thomas G L, Spandl R J, Glansdorp F G, Welch M, Bender A, Cockfield J, Lindsay J A, Bryant C, Brown D F J, Loiseleur O (2008). Angew Chem, Int Ed.

[31] Hall H K (1957). J Am Chem Soc.

