# Supplementary material for: Two-directional synthesis as a tool for diversity-oriented synthesis: Synthesis of alkaloid scaffolds
Source: Beilstein J Org Chem. 2012 Jun 6;8:850–60. doi: 10.3762/bjoc.8.95 (PMC3388873; doi:10.3762/bjoc.8.95)
Supplement: File 1 — Experimental procedures and spectral data for all previously unreported compounds. [file Beilstein_J_Org_Chem-08-850-s001.pdf]

Supporting Information

for

Two-directional synthesis as a tool for diversity-oriented synthesis: Synthesis of alkaloid scaffolds

Kieron M. G. O'Connell[1], Monica Díaz-Gavilán[1,§], Warren R. J. D. Galloway[1] and David R. Spring*[1]

Address: [1]Department of Chemistry, University of Cambridge, Lensfield Rd, Cambridge, CB2 1EW, UK

[§]Present address: Departemento de Química Farmacéutica y Orgánica, Facultad de Farmacia, Campus de Cartuja, s.n. 18071 Granada, Spain

Email: David R. Spring - spring@ch.cam.ac.uk

* Corresponding author

Experimental procedures and spectral data for all previously unreported compounds

Table of Contents

1. General experimental details S2
2. Synthesis of *N*-Boc-aminodialkenes S3
3. Cyclisation reactions of amino alkenes S12
4. Total synthesis of myrrhine S17
5. Alternative starting materials S18

1. General experimental details

Reactions were performed using oven-dried glassware under an atmosphere of nitrogen with anhydrous, freshly distilled solvents only when indicated. Dichloromethane, ethyl acetate, methanol and *n*-hexane were distilled from calcium hydride. Anhydrous dimethylformamide (DMF) was used as obtained from commercial sources. All other reagents were used as obtained from commercial sources.

Room temperature refers to ambient temperature. Temperatures of 0 °C were maintained by using an ice–water bath and temperatures below 0 °C were maintained using an acetone–cardice bath

Yields refer to chromatographically and spectroscopically pure compounds unless otherwise stated. All flash chromatography was carried out using slurry-packed Merck 9325 Kieselgel 60 silica gel. Where possible, reactions were monitored by thin layer chromatography (TLC) performed on commercially prepared glass plates precoated with Merck silica gel 60 F_{254} or aluminium oxide 60 F_{254}. Visualisation was by the quenching of UV fluorescence (v_{max} = 254 nm) or by staining with ceric ammonium molybdate, potassium permanganate, iodine or Dragendorff's reagent (0.08% w/v bismuth subnitrate and 2% w/v KI in 3M aq. AcOH).

Infrared spectra were recorded on a Perkin-Elmer Spectrum One spectrometer with internal referencing. Selected absorption maxima (v_{max}) are reported in wavenumbers (cm^{-1}).

Melting points were obtained using a Büchi® melting point apparatus (model B-545) and are uncorrected.

Proton magnetic resonance spectra were recorded by using an internal deuterium lock at ambient probe temperatures on the following instruments: Bruker DPX-400 (400 MHz), Bruker Avance 400 QNP (400 MHz), Bruker Avance 500 BB ATM (500 MHz) and Bruker Avance 500 Cryo Ultrashield (500 MHz). Chemical shifts (δ_H) are quoted in ppm, to the nearest 0.01 ppm, and are referenced to the residual nondeuterated solvent peak. Coupling

constants (*J*) are reported in Hertz to the nearest 0.5 Hz. Data are reported as follows: chemical shift, multiplicity [b, broad; s, singlet; d, doublet; t, triplet; q, quartet; quint, quintet; sept, septet; m, multiplet; or as a combination of these (e.g., dd, dt, etc.)], coupling constant(s), integration and assignment. Proton assignments were determined either on the basis of unambiguous chemical shift or coupling pattern, by patterns observed in 2D experiments (^1H–^1H COSY, HMBC and HMQC) or by analogy to fully interpreted spectra for related compounds. NOESY experiments were used to determine the stereochemistry when necessary. Numbering of compounds for NMR assignment does not correspond to the numbering for nomenclature.

Carbon magnetic resonance spectra were recorded by broadband proton spin decoupling at ambient probe temperatures by using an internal deuterium lock on the following instruments: Bruker DPX-400 (100 MHz), Bruker Avance 400 QNP (100 MHz), Bruker Avance 500 BB ATM (125 MHz) and Bruker Avance 500 Cryo Ultrashield (125 MHz). Chemical shifts (δ_C) are quoted in ppm, to the nearest 0.1 ppm, and are referenced to the residual nondeuterated solvent peak. Assignments were supported by DEPT editing and determined either on the basis of unambiguous chemical shift, by patterns observed in 2D experiments (HMBC and HMQC) or by analogy to fully interpreted spectra for related compounds.

High-resolution mass spectroscopy measurements were made by the EPSRC mass spectrometry service (Swansea) or recorded in-house by using a Waters LCT Premier Mass Spectrometer or a Micromass Quadrapole-Time of Flight (Q-ToF) spectrometer. Mass values are reported within the error limits of ±5 ppm mass units. ESI = electrospray ionisation.

2. Synthesis of *N*-Boc-aminodialkenes

Full details for the synthesis and characterization of the following compounds can be found in our previous communication [1]

Nona-1,8-dien-5-ol [1]

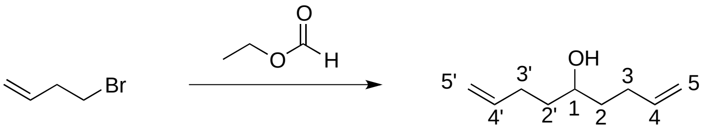

Deca-1,9-dien-5-ol [1]

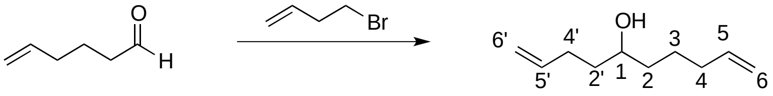

Undeca-1,10-dien-6-ol [1]

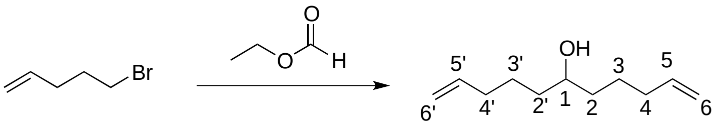

5-Phenylnona-1,8-dien-5-ol

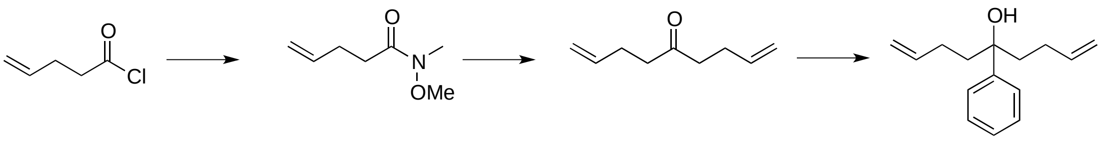

4

***N*-Methoxy-*N*-methyl-4-pentenamide** [2]

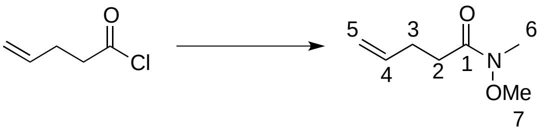

A solution of 4-pentenoyl chloride (1.0 equiv) in anhydrous dichloromethane (9 mL/mmol of 4-pentenoyl chloride) was prepared under nitrogen, at rt. To this solution, solid *N*,*O*-dimethylhydroxyamine (1.1 equiv) was added and the mixture was then cooled to 0 °C for the addition of anhydrous pyridine (2.2 equiv). After this, the reaction was warmed up to rt and stirred for 1.5 h. The solvent was then evaporated and the residue was partitioned between brine and a 1:1 mixture of diethyl ether and dichloromethane. The organic layer was

dried over magnesium sulfate, filtered and evaporated under vacuum. The residue was purified by flash chromatography on silica eluting with a mixture of dichloromethane/methanol 10:0.4, to furnish the final Weinreb amide as a yellow oil in quantitative yield: R_f 0.43 (petroleum ether (30-40)/diethyl ether 1:1).

1,8-Nonadien-5-one [3]

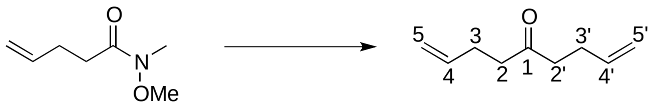

A suspension of magnesium turnings (2.5 equiv) and iodine (cat.) in dry THF (2 mL/mmol of bromide) was prepared at rt, under a nitrogen atmosphere. To this mixture, 4-bromo-1-butene (2.5 equiv) was added slowly at rt. During the addition, an increase in the temperature of the reaction mixture confirmed the initiation of the Grignard formation. Once the addition of the bromide was complete, the mixture was stirred at rt for 45 min, after which it was transferred to an addition funnel connected to a flask containing a solution of *N*-methoxy-*N*-methyl-4-pentenamide (1.0 equiv) in anhydrous THF (4 mL/mmol), under stirring at −10 °C, in a nitrogen atmosphere. The solution of the Grignard was added dropwise, after which the mixture was allowed to warm up to rt and stirred for 24 h. After this time the reaction was quenched by cooling to 0 °C and addition of aqueous ammonium chloride (saturated solution). The aqueous phase was extracted with diethyl ether (×3), the mixed organic layers were dried over magnesium sulfate, filtered and evaporated under vacuum. The residue was purified by flash chromatography on silica eluting with a mixture of petroleum ether (30/40)/diethyl ether 20:1, to furnish the desired ketone as a yellow oil in 81% yield [R_f 0.50 (petroleum ether (30-40)/diethyl ether 19:1)] ν_{max} (CH_2Cl_2/cm^{-1}) 3079, 2923, 1715, 1641, 1414, 908; δ_H (400 MHz; $CDCl_3$) 5.80 (ddt, J_1 = 16.8 Hz, J_2 = 10.2 Hz, J_3 = 6.5 Hz, 2H, H-4, 4’), 5.01 (dq, J_1 = 17.0 Hz, J_2 = 1.6 Hz, 2H, H_{trans}-5_A, 5’$_A$), 4.96 (dq, J_1 = 10.2 Hz, J_2 = 1.3 Hz, 2H, H_{cis}-5_B, 5’$_B$), 2.50 (t, J = 7.3 Hz, 4H, H-2, 2’), 2.31 (q, 4H, H-3, 3’); δ_C (100 MHz; $CDCl_3$) 209.3 (C-1), 137.1 (CH-4 and 4’), 115.2 (CH_2-5 and 5’), 41.9 ($2CH_2$), 27.7 ($2CH_2$).

5-Phenylnona-1,8-dien-5-ol

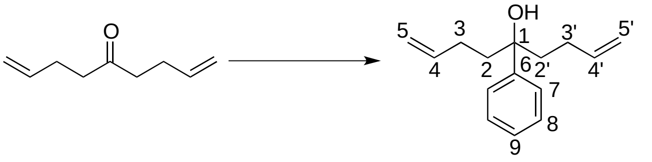

A solution of phenylmagnesium bromide (2.2 M in diethyl ether, 1.1 equiv) in anhydrous THF (5 mL/mmol of Grignard) was prepared under nitrogen, at −5 °C. To this solution, 1,8-nonadien-5-one was added dropwise at the same temperature and the mixture was stirred for 3.5 h, at between −5 and 0 °C. After this time, no starting material was observed on TLC and the reaction was quenched by acidification with hydrochloric acid (3 N). The aqueous phase was extracted with ethyl acetate (×3) and the combined organic phases were dried over magnesium sulfate, filtered and evaporated under vacuum. The residue was purified by flash chromatography on silica (stepped gradient elution with mixtures petroleum ether (30/40)/diethyl ether 20:1, 16:1 and 14:1), to furnish **4** as a yellow oil in 73% yield [R_f 0.26 (petroleum ether (30-40)/diethyl ether 15:1)] ν_{max} (CH_2Cl_2/cm^{-1}) 3032, 2922, 1711, 1640, 1481, 1446, 1431, 1008, 909, 737, 729, 700; δ_H (400 MHz; $CDCl_3$) 7.39–7.33 (m, 4H, H-7, 7’, 8, 8’), 7.26–7.22 (m, 1H, H-9), 5.79 (ddt, J_1 = 16.8 Hz, J_2 = 10.2 Hz, J_3 = 6.4 Hz, 2H, H-4, 4’), 4.95 (pdd, J_1 = 17.1 Hz, J_2 = 1.5 Hz, 2H, H_{trans}-5_A, $5’_A$), 4.92 (pd, J = 10.2 Hz, 2H, H_{cis}-5_B, $5’_B$), 2.09–2.04 (m, 2H), 1.98–1.83 (m, 6H); δ_C (100 MHz; $CDCl_3$) 145.6 (C-6), 138.8 (CH-4 and 4’), 128.1, 125.2 (CH_2-7, 7’, 8 and 8’), 126.4 (CH-9), 114.6 (CH_2-5 and 5’), 77.2 (C-1), 42.1 ($2CH_2$), 28.0 ($2CH_2$); HRMS (ESI^+) calcd for $C_{15}H_{21}O$ $(M + H)^+$ 217.1592, found 217.1019.

Full details for the synthesis and characterization of the following compounds can be found in our previous communication [1].

***tert*-Butyl *N*-(*p*-toluenesulfonyl)-*N*-[1-(3-butenyl)-4-pentenyl]carbamate** [1]

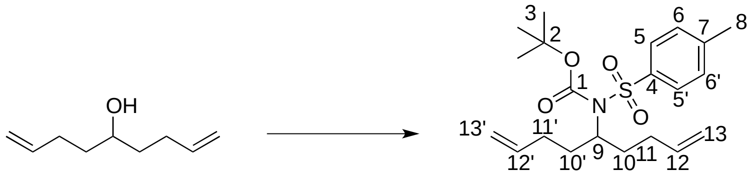

***tert*-Butyl *N*-(*p*-toluenesulfonyl)-*N*-[1-(3-butenyl)-5-hexenyl]carbamate** [1]

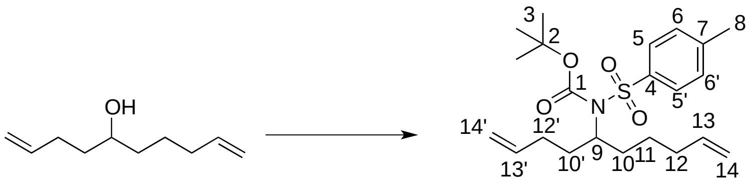

***tert*-Butyl *N*-(*p*-toluenesulfonyl)-*N*-[1-(4-pentenyl)-5-hexenyl]carbamate** [1]

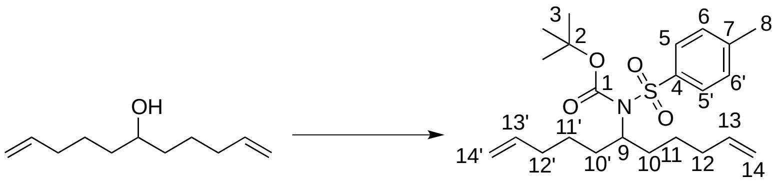

***tert*-Butyl *N*-[1-(3-butenyl)-4-pentenyl]carbamate** [1]

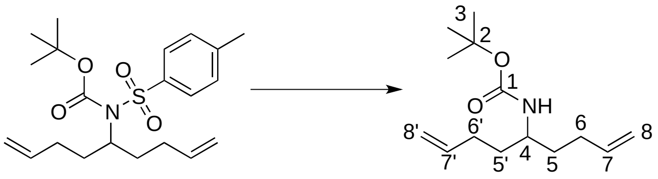

***tert*-Butyl *N*-[1-(3-butenyl)-5-hexenyl]carbamate** [1]

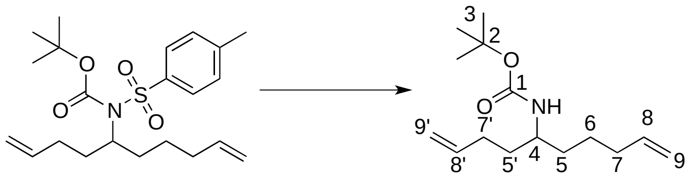

***tert*-Butyl *N*-[1-(4-pentenyl)-5-hexenyl]carbamate** [1]

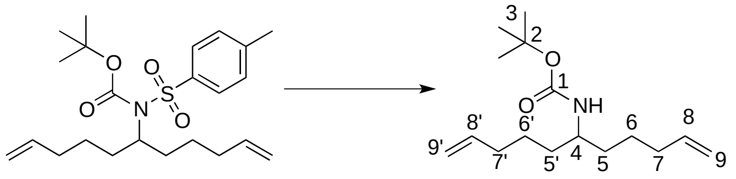

(2*E*, 9*E*)-Diethyl 6-(*tert*-butoxycarbonylamino)undeca-2,9-dienedioate (1) [1]

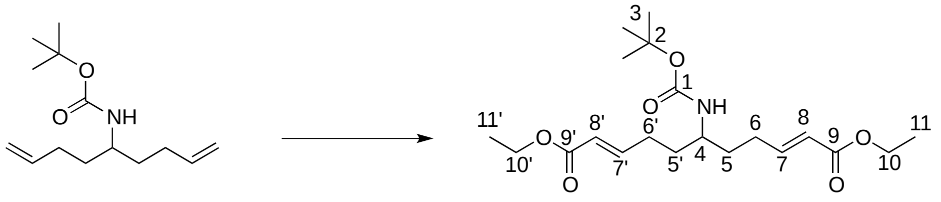

(2*E*, 10*E*)-Diethyl 6-(*tert*-butoxycarbonylamino)dodeca-2,10-dienedioate (2) [1]

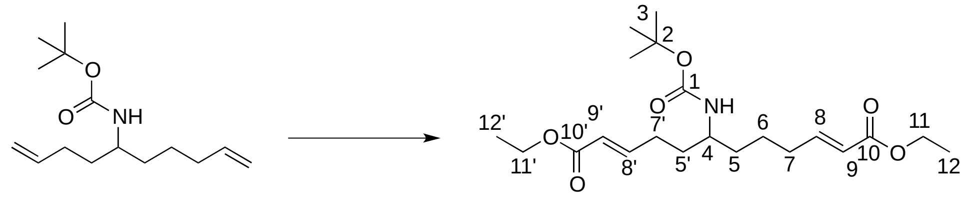

(2*E*, 11*E*)-Diethyl 7-(*tert*-butoxycarbonylamino)trideca-2,11-dienedioate (3) [1]

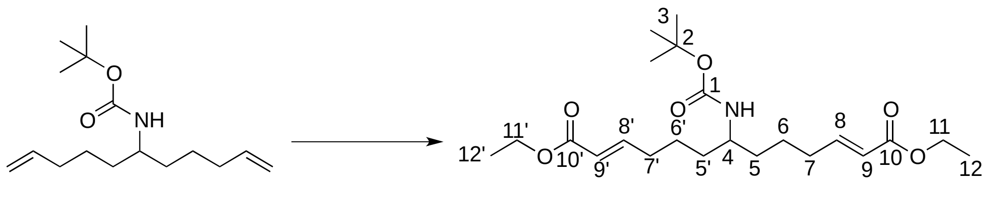

2-Chloro-*N*-(5-phenylnona-1,8-dien-5-yl)acetamide

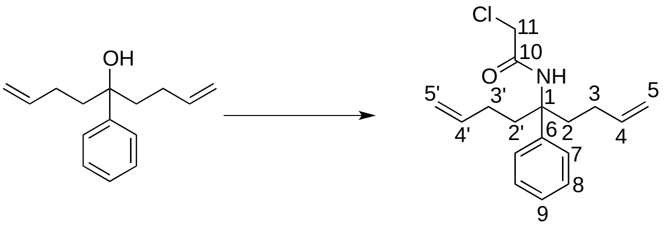

A mixture of the alcohol (1.0 equiv) and chloroacetonitrile (49 equiv) was stirred at rt. To this mixture, glacial acetic acid (0.06 mL/mmol of alcohol) was added before cooling to 0 °C. At this temperature, 98% sulfuric acid (1.0 equiv) was dropped and the resulting mixture was allowed to warm up to rt [4]. Once at rt, the mixture was stirred for 5 h. The reaction was then stopped by partition between water and diethyl ether and extraction of the aqueous phase with diethyl ether (×3). The final organic phase was washed with sodium bicarbonate (saturated solution) and brine. It was then dried over magnesium sulfate, filtered and evaporated under vacuum. The residue was purified by flash chromatography on silica (stepped gradient elution with mixtures petroleum ether (30/40)/diethyl ether 18:1, 12:1 and 8:1), to furnish the title compound as a white solid in 68% yield [mp 113–114 °C (petroleum ether/diethyl ether); R_f 0.27 (petroleum ether (30-40)/diethyl ether 5:1)] ν_{max} (CH_2Cl_2/cm^{-1}) 3283, 3077, 1667, 1558, 1238, 913; δ_H (400 MHz; $CDCl_3$) 7.39–7.35 (m, 2H, H_{Ar}), 7.32–7.25 (m, 3H, H_{Ar}), 6.83 (bb, 1H, N*H*), 5.75 (ddt, J_1 = 16.8 Hz, J_2 = 10.2 Hz, J_3 = 6.5 Hz, 2H, H-4, 4’), 4.97 (pdd, J_1 = 17.1 Hz, J_2 = 1.6 Hz, 2H, H_{trans}-5_A, $5’_A$), 4.93 (pdd, J_1 = 10.2 Hz, J_2 = 1.3 Hz, 2H, H_{cis}-5_B, $5’_B$), 4.03 (s, 2H, H-11), 2.33–2.18 (m, 4H), 1.87 (m, 4H); δ_C (100 MHz; $CDCl_3$) 164.6 (C-10), 143.2 (C-6), 138.0 (CH-4 and 4’), 128.8, 125.4 (CH_2-7, 7’, 8 and 8’), 127.2 (CH-9), 115.0 (CH_2-5 and 5’), 62.5 (C-1), 43.2, 36.9, 28.2 (CH_2); HRMS (ESI^+) calcd for $C_{17}H_{23}ClNO$ $(M + H)^+$ 292.1468, found 292.1462.

5-Phenylnona-1,8-dien-5-amine [5]

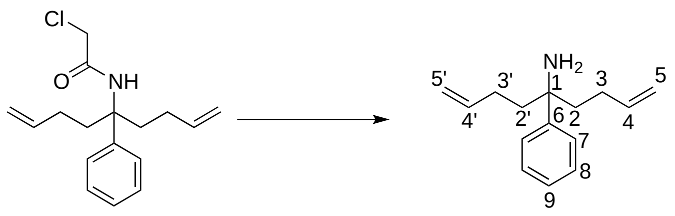

A mixture of the amide 2-chloro-*N*-(5-phenylnona-1,8-dien-5-yl)acetamide (1.0 equiv), thiourea (1.2 equiv) and ethanol/acetic acid 5:1 (2.4 mL/mmol) was prepared at rt and then heated up to reflux and stirred during 20 h. The mixture was then cooled to rt and diluted with water. The resulting mixture was filtered and the filtrate was basified with NaOH (1 M in H_2O). The aqueous phase was extracted with petrol ether (30/40) (×3) and the resulting organic phase was dried over magnesium sulfate, filtered and evaporated to furnish the title compound (yellow oil, 95%) [R_f 0.40 (petroleum ether (30-40)/ethyl acetate 1:2)] v_{max} (CH_2Cl_2/cm^{-1}) 3076, 2924, 1639, 1446, 1205, 1184, 1139, 910; δ_H (400 MHz; $CDCl_3$) 7.72 (m, 2H, H-7, 7'), 7.36 (m, 2H, H-8, 8'), 7.24 (ddt, J_1 = J_2 = 7.1 Hz, J_3 = 1.3 Hz, 1H, H-9), 5.77 (ddt, J_1 = 16.8 Hz, J_2 = 10.0 Hz, J_3 = 7.0 Hz, 2H, H-4, 4'), 4.97 (dd, J_1 = 17.0 Hz, J_2 = 2.0 Hz, 2H, H_{trans}-5_A, $5'_A$), 4.92 (dd, J_1 = 10.0 Hz, J_2 = 1.6 Hz, 2H, H_{cis}-5_B, $5'_B$), 2.06–1.91 (m, 4H), 1.85–1.80 (m, 4H).

***tert*-Butyl *N*-(5-phenylnona-1,8-dien-5-yl)carbamate**

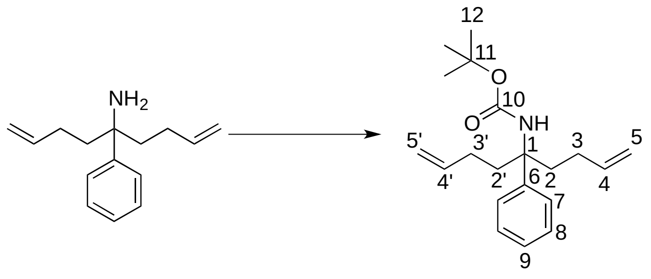

A solution of $(Boc)_2O$ (1.5 equiv) in anhydrous acetonitrile (2 mL/mmol) was prepared under a nitrogen atmosphere. Into this solution, a solution of 5-phenylnona-1,8-dien-5-amine in anhydrous acetonitrile (6 mL/mmol) was added [6].The mixture was stirred at rt for 24 h after which time the solvent was removed under vacuum and the residue was purified by flash

chromatography on silica (stepped gradient elution with mixtures petroleum ether (30/40)/diethyl ether 30:1, 20:1 and 16:1), to furnish the title compound as a white solid in 74% yield [mp 73–74 °C (petroleum ether/diethyl ether); R_f 0.48 (petroleum ether (30-40)/diethyl ether 10:1)] ν_{max} (CH_2Cl_2/cm^{-1}) 3273, 2975, 1727, 1685, 1485, 1365, 1245, 1166, 909; δ_H (400 MHz; $CDCl_3$) 7.35 (m, 4H, H_{Ar}), 7.27–7.22 (m, 1H, H-9), 5.79 (ddt, J_1 = 17.5 Hz, J_2 = 10.0 Hz, J_3 = 6.7 Hz, 2H, H-4, 4'), 4.99 (bd, J = 17.5 Hz, 2H, H_{trans}-5_A, $5'_A$), 4.94 (bd, J = 10.0 Hz, 2H, H_{cis}-5_B, $5'_B$), 4.85 (bb, 1H, N*H*), 2.20–2.07 (m, 4H), 2.00–1.85 (m, 4H), 1.42 (bb, 9H); δ_C (100 MHz; $CDCl_3$) 155.0 (C-10), 138.7, 128.6, 126.9 (CH_{Ar}), 126.0 (CH-4 and 4'), 115.0 (CH_2-5 and 5'), 79.5 (C-11), 61.0 (C-1), 37.8 (CH_2), 28.7 (CH_2), 28.5 (CH_2); HRMS (ESI^+) calcd for $C_{20}H_{29}NNaO_2$ (M + Na^+) 338.2096, found 338.2120.

(2*E*,9*E*)-Diethyl 6-(*tert*-butoxycarbonylamino)-6-phenylundeca-2,9-dienedioate (4)

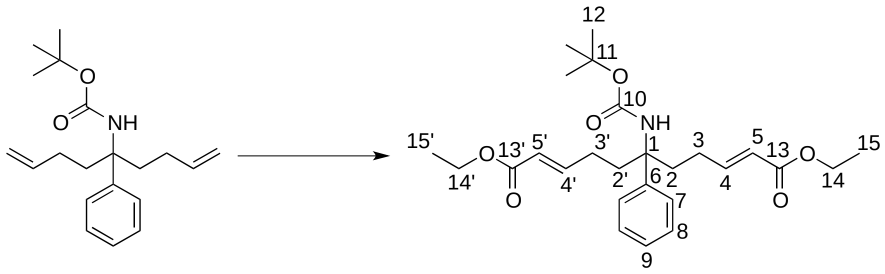

In a dry flask, a 0.08 M solution of *tert*-butyl *N*-(5-phenylnona-1,8-dien-5-yl)carbamate (1.0 equiv) in ethyl acrylate was prepared and degassed (nitrogen bubbling during 10–15 min), at rt. To the degassed solution, Grubbs second generation catalyst (3 mol %) was added as a solid, and nitrogen was again bubbled in the reaction mixture for 10 min more. After this time, the mixture was stirred under a nitrogen atmosphere for 24 h. The reaction crude was then loaded directly in a column for removal of the ethyl acrylate and purification of the final product. Gradient elution with petroleum ether (30/40)/diethyl ether from 10:1 to 2:1, afforded the title compound [67%, colourless oil, R_f 0.20 (petroleum ether (30/40)/diethyl ether 2:1)]. ν_{max} (CH_2Cl_2/cm^{-1}) 3357, 2977, 1716, 1652, 1494, 1366, 1264, 1164, 1042; δ_H (400 MHz; $CDCl_3$) 7.39–7.25 (m, 5H, H_{Ar}), 6.89 (dt, J_{trans} = 15.7 Hz, J_2 = 7.0

Hz 2H, H-4, 4'), 5.76 (bd, J_{trans} = 15.6 Hz, 2H, H-5, 5'), 4.92 (bb, 1H, N*H*), 4.17 (q, *J* = 7.3 Hz, 4H, H-14, 14'), 2.31 (bb, 2H), 2.20–1.95 (m, 6H), 1.43 (bb, 9H, H-12), 1.29 (t, *J* = 7.1 Hz, 6H, H-15, 15'); δ_C (100 MHz; $CDCl_3$) 166.8 (C-13 and 13), 154.5 (C-10), 143.7 (C-6), 148.5, 129.0, 127.4 125.7, 122.0 (3CH_{Ar}, CH-4, 4´, 5 and 5'), 80.0 (C-11), 60.7 (C-1), 60.6 (CH_2-14 and 14'), 36.9, 27.1 (CH_2-2, 3, 2' and 3'), 28.7 (CH_3-12), 14.6 (CH_3-15); HRMS (ESI^+) calcd for $C_{26}H_{37}NNaO_6$ (M + Na^+) 482.2519, found 482.2545.

3. Cyclisation reactions of amino alkenes

(3*R*,5*R*)/(3*S*,5*S*)-Diethyl 2,2'-(hexahydro-1*H*-pyrrolizin-3,5-diyl)diacetate [7] **(*trans*-5) and (3*R*,5*S*,7a*r*)-diethyl 2,2'-(hexahydro-1*H*-pyrrolizin-3,5-diyl)diacetate (*cis*-5)**

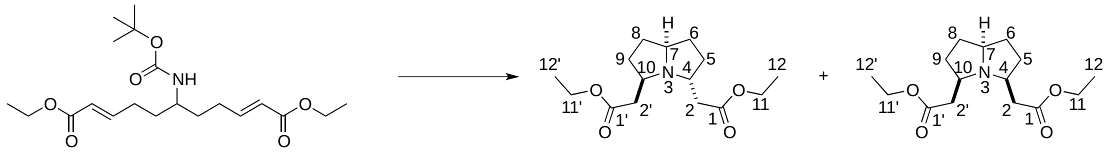

Full details for the synthesis and characterization of these compounds can be found in our previous communication [1].

(3*R*,5*R*)/(3*S*,5*S*)-Diethyl 2,2'-(7a-phenylhexahydro-1*H*-pyrrolizine-3,5-diyl)diacetate (*trans-6*) and (3*R*,5*S*,7a*s*)-diethyl 2,2'-(7a-phenylhexahydro-1*H*-pyrrolizine-3,5-diyl)diacetate (***cis-6***)

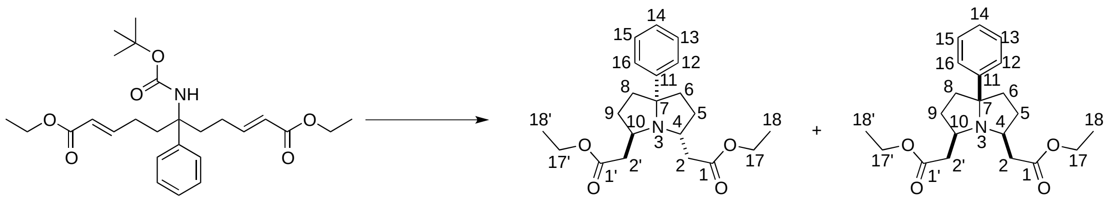

In a dry flask, a solution of **2** (1.0 equiv) in anhydrous dichloromethane (30mL/mmol of **2**) was prepared under nitrogen and cooled to 0 °C. At this temperature, aluminium chloride (1.0 M in nitrobenzene) was added dropwise (1.1 equiv). After 10 min at low temperature, the reaction mixture was warmed

up to rt and stirred for 24 h. The reaction was quenched by addition of sodium bicarbonate (5% solution in water) and separation of the phases. The organic phase was washed with water, dried over magnesium sulfate, filtered and evaporated. The residue was purified by column chromatography. Nitrobenzene was removed by elution with petroleum ether (30/40)/diethyl ether 10:1. Posterior elution with petroleum ether (30/40)/diethyl ether 4:1 afforded a mixture of both isomers in proportions 3:1 ***trans***:***cis*** [colourless oil, R_f 0.45 (petroleum ether (30/40)/diethyl ether 6:1); ν_{max} (CH_2Cl_2/cm^{-1}) 2961, 2870, 1728, 1445, 1369, 1274, 1248, 1164, 1029, 765, 705; HRMS (ESI^+) calcd for $C_{21}H_{30}NO_4$ $(M + H)^+$ 360.2175, found 360.2208]. The separation of both isomers was done on elution with DCM to extract ***cis-6*** [17%, colourless oil, R_f 0.27 (DCM)], followed by DCM:diethyl ether mixtures 10:0.25 and 10:0.5 to extract ***trans-6*** [50%, colourless oil, R_f 0.20 (DCM)].

cis-19: δ_H (400 MHz; $CDCl_3$) 7.53–7.50 (m, 2H, H-12, 16), 7.30–7.27 (m, 2H, H-13, 15), 7.20–7.17 (m, 1H, H-14), 4.19 (dq, J_{gem} = 11.2 Hz, J_{vec} = 7.2 Hz, 2H, H-17_A, 17'$_A$), 4.14 (dq, J_{gem} = 11.2 Hz, J_{vec} = 7.2 Hz, 2H, H-17_B, 17'$_B$), 3.40 (bquint, J = 6.4 Hz, 2H, H-4, 10), 2.64 (dd, J_{gem} = 15.0 Hz, J_{vec} = 5.7 Hz, 2H, H-2_A, 2'$_A$), 2.29 (dd, J_{gem} = 15.0 Hz, J_{vec} = 7.8 Hz, 2H, H-2_B, 2'$_B$), 2.10–1.95 (m, 6H, H-5_A, 6_A, 8_A, 9_A, 6_B, 8_B), 1.50–1.42 (m, 2H, 5_B, 9_B), 1.29 (t, J = 7.1 Hz, 6H, H-18, 18'); δ_C (125 MHz; $CDCl_3$) 172.5 (C-1 and 1'), 152.2 (C-11), 128.1 (CH-13 and 15), 126.3 (CH-12 and 16), 126.1 (CH-14), 77.6 (C-7), 64.8 (CH-4 and 10), 60.5 (CH_2-17 and 17'), 41.8 (CH_2-2 and 2'), 41.0 (CH_2-6 and 8), 30.9 (CH_2-5 and 9), 14.6 (CH_3-18 and 18').

trans-6: δ_H (400 MHz; $CDCl_3$) 7.54–7.52 (m, 2H, H-12, 16), 7.32–7.27 (m, 2H, H-13, 15), 7.24–7.17 (m, 1H, H-14), 4.24–4.13 (m, 4H, H-17 and 17'), 3.67 (bquint, J = 5.0 Hz, 1H, H-10), 3.61 (bquint, J = 7.1 Hz, 1H, H-4), 2.99 (dd, J_{gem} = 14.6 Hz, J_{vec} = 5.0 Hz, 1H, H-2'$_A$), 2.63 (dd, J_{gem} = 14.4 Hz, J_{vec} = 5.8 Hz, 1H, H-2_A), 2.38 (dd, J_{gem} = 15.0 Hz, J_{vec} = 7.5 Hz, 1H, H-2_B), 2.34 (dd, J_{gem} = 14.4 Hz, J_{vec} = 9.6 Hz, 1H, H-2'$_B$), 2.19–2.14 (m, 1H, H-6_A), 2.10–1.85 (m, 6H, H-5_A, 8_A, 9_A, 5_B, 6_B, 8_B), 1.66–1.60 (m, 1H, H-9_B), 1.31–1.27 (m, 6H, H-18, 18'); δ_C (125 MHz; $CDCl_3$) 172.5 (C-1 and 1'), 151.0 (C-11), 128.2 (CH-13 and 15), 126.4 (CH-12 and 16), 126.2 (CH-14), 78.2 (C-7), 60.9, 60.7

(CH_2-17 and 17’), 57.9 (CH-10), 55.5 (CH-4), 43.0 (CH_2-2), 41.9 (CH_2-6), 39.9 (CH_2- 8), 37.0 (CH_2-2’), 34.0 (CH_2-5), 32.6 (CH_2- 9), 14.6 (CH_3-18 and 18’).

(3*R*,5*R,*8a*R*)/(3*S*,5*S,*8a*S*)-Diethyl 2,2’-(octahydroindolizine-3,5-diyl)diacetate, (*trans*-7) and **(3*S*,5*S,*8a*R*)**/**(3*R*,5*R,*8a*S*)-diethyl 2,2’-(octahydroindolizine-3,5-diyl)diacetate, (*trans’*-7)**

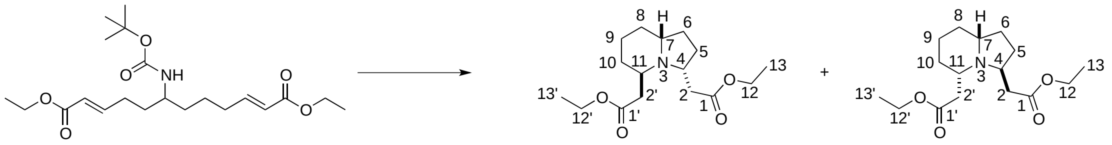

Full details for the synthesis and characterization of these compounds can be found in our previous communication [1].

(*E*)-Ethyl 6-((2*R*,6*R*)/(2*S*,6*S*)-6-(2-ethoxy-2-oxoethyl)piperidin-2-yl)hex-2-enoate (9)

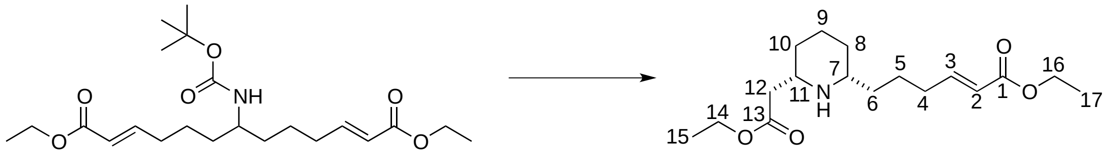

A solution of **3** (1.0 equiv) in dry dichloromethane (30 mL/mmol of **3**) was prepared at rt. After cooling down to 0 °C, 1.1 equiv of aluminium chloride (1 M in nitrotoluene) were added. Once the addition was completed, the cold bath was removed and the mixture was stirred at rt for 24 h. After this time, the reaction was cooled to 0 °C and diluted with dichloromethane and sodium bicarbonate (saturated aqueous solution). The phases were separated and the organic phase was washed with water, dried on magnesium sulfate, filtered and evaporated under vacuum. The residue was purified by flash chromatography on silica. First, elution with mixtures petroleum ether (30/40)/diethyl ether 2:1→1.5:1, afforded ***trans-8*** as a colorless oil in 8% yield and later elution with mixtures ethyl acetate/acetone 1:1 and 0:1 afforded the monocycle **9** in 40% yield [R_f 0.27 (ethyl acetate/acetone 2:1)] ν_{max} (CH_2Cl_2/cm^{-1}) 3351, 2968, 2927, 2845, 2346, 2129, 1690 (bb), 1521, 1455, 1366, 1247, 1174, 909; δ_H (400 MHz; CDCl3) 6.94 (dt, J_1 = 15.6 Hz, J_2 = 7.0

Hz, 1H, H-3), 5.81 (dt, J_1 = 15.6 Hz, J_2 = 1.4 Hz, 1H, H-2), 4.18 (q, J = 7.2 Hz, 2H, H-14 or 16), 4.14 (dt, J = 7.2 Hz, 2H, H-14 or 16), 2.93 (m, 1H, H-11), 2.56–2.52 (m, 1H, H-7), 2.40 (dd, J_1 = 15.8 Hz, J_2 = 4.6 Hz, 1H, H-12A), 2.36 (dd, J_1 = 15.6 Hz, J_2 = 8.2 Hz, 1H, H-12B), 2.21 (qd, J_1 = 7.2 Hz, J_2 = 1.6 Hz, 2H, H-4), 1.79 (m, 1H, H-6A), 1.67–1.56 (m, 2H, H-8A, 10A), 1.56–1.45 (m, 2H, H-5), 1.44–1.36 (m, 1H, H-9A), 1.36–1.32 (m, 1H, H-6B), 1.32–1.28 (m, 1H, H- 9B), 1.28 (t, J = 7.2 Hz, 3H, H-15 or 17), 1.26 (t, J = 7.2 Hz, 3H, H-15 or 17), 1.16–1.07 (m, 1H, H-10B), 1.07–0.80 (m, 1H, H-8B); δ_C (125 MHz; $CDCl_3$) 172.5 (C-13), 166.4 (C-1), 148.8 (CH-3), 121.6 (CH-2), 60.4 (CH2-14), 60.1 (CH2-16), 56.5 (CH-7), 53.4 (CH-11), 41.4 (CH2-12), 36.7 (CH2-9), 32.3 (CH2-10), 32.2 (CH2-4), 32.0 (CH2-8), 24.5 (CH2-6), 24.4 (CH2-5), 14.3, 14.2 (CH3-15 and 17).

(4*R*,6*R*)/(4*S*,6*S*)-Diethyl 2,2'-(octahydro-1*H*-quinolizine-4,6-diyl)diacetate (*trans-8*)

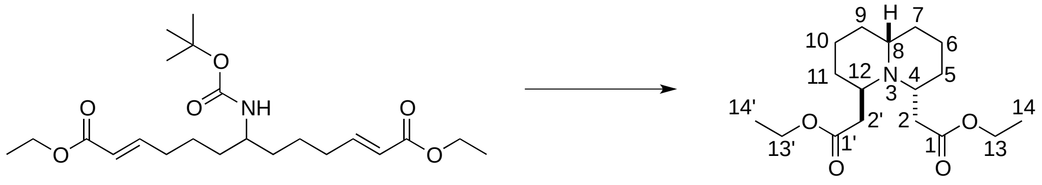

Full details for the synthesis and characterization of this compound can be found in our previous communication [1].

(1*R*, 3a*S*, 6a*R*, 9a*R*)/(1*S*, 3a*R*, 6a*S*, 9a*S*)-Ethyl 2-oxododecahydropyrido[2,1,6-*de*]quinolizine-1-carboxylate (10) diethyl 2,2'-((4R,6S,9ar)-octahydro-1H-quinolizine-4,6-diyl)diacetate (*cis*-8)

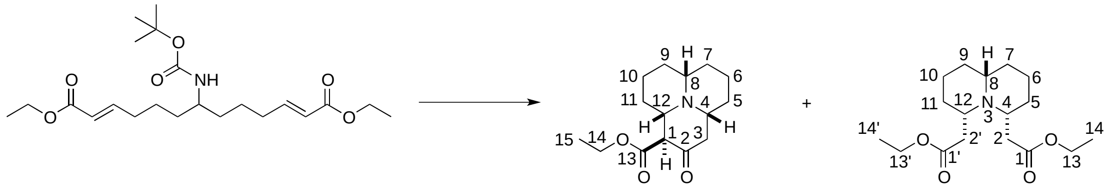

Full details for the synthesis and characterization of these compounds can be found in our previous communication [1].

(2a*R*, 5*S*, 5a*S*, 9a*R*)/(2a*S*, 5*R*, 5a*R*, 9a*S*)-Ethyl 4-oxododecahydro-1*H*-pyrrolo[2,1,5-*de*]quinolizine-5-carboxylate (*trans*-13) and (2a*R*, 3*S*, 5a*R*, 5a*R*)/ (2a*S*, 3*R*, 5a*S*, 5a*S*)-ethyl 4-oxodecahydro-1*H*-pyrrolo[2,1,5-*de*]quinolizine-3-carboxylate (*cis*-13)

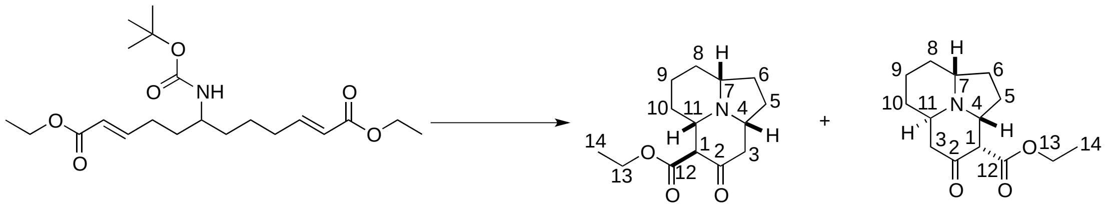

Full details for the synthesis and characterization of these compounds can be found in our previous communication [1].

(2a*S*,4a*S*,7a*R*)/(2a*R*,4a*R*,7a*S*)-Ethyl 6-hydroxy-1,2,2a,3,4,4a,7,7a-octahydropyrrolo [2,1,5-*cd*]indolizine-5-carboxylate (***cis*-14**)

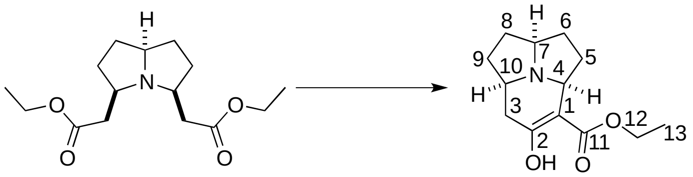

Full details for the synthesis and characterization of these compounds can be found in our previous communication [1].

(2a*R*,4a*R*,7a*R*)/(2a*S*,4a*S*,7a*S*)-Ethyl 6-hydroxy-1,2,2a,3,4,4a,7,7a-octahydropyrrolo [2,1,5-*cd*]indolizine-5-carboxylate (***trans*-14**)

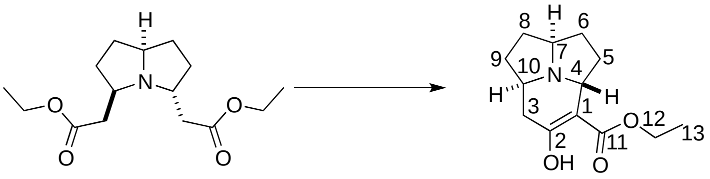

Full details for the synthesis and characterization of these compounds can be found in our previous communication [1].

4. Total synthesis of myrrhine

Full details for the synthesis and characterization of the following compounds can be found in our previous communication [1].

Decahydro-1*H*-pyrido[2,1,6-*de*]quinolizin-2-one [1]

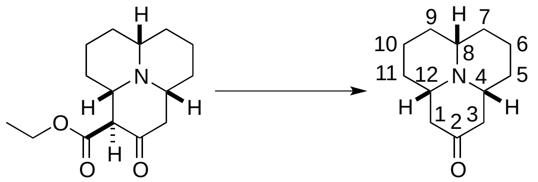

2-Methylenedodecahydropyrido[2,1,6-*de*]quinolizine (15) [1]

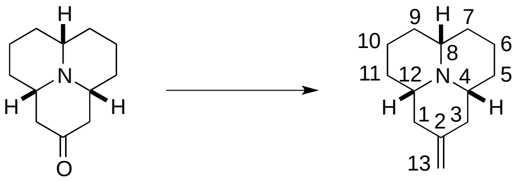

(2*r*, 3a*R*, 7a*r*, 10a*S*)-2-Methyldodecahydropyrido-1*H*-[2,1,6-*de*]quinolizine, myrrhine (and *epi*-myrrhine) [1,8]

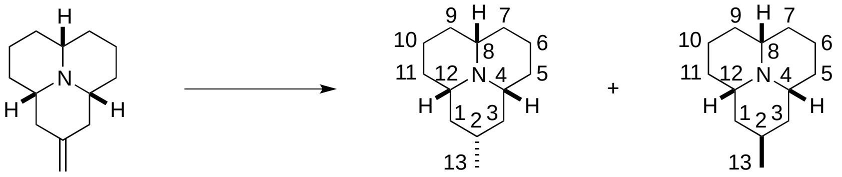

(2*r*, 3a*R*, 7a*r*, 10a*S*)-2-Methyl dodecahydro-1*H*-pyrido[2,1,6-*de*]quinolizine-4-oxide myrrhine-*N*-oxide [1]

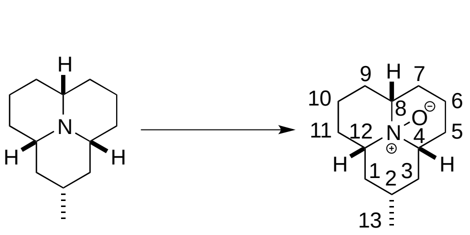

5. Alternative starting materials

Synthesis of *meso* 3,5-diphenylpyrrolizidine (17)

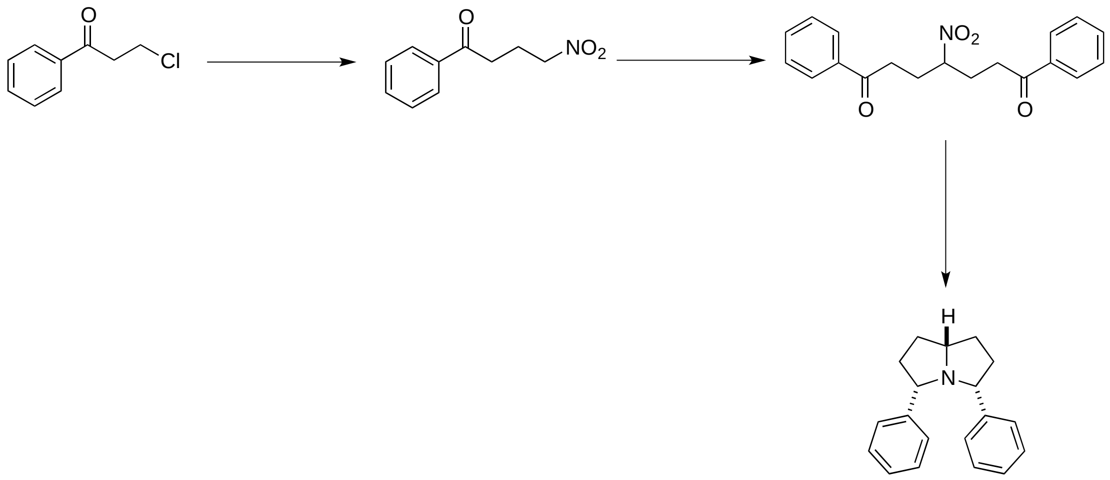

4-Nitrobutyrophenone [9]

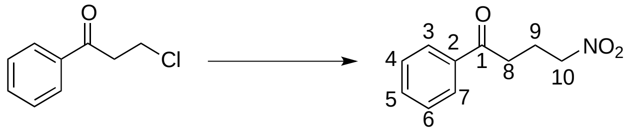

To a stirred solution of nitromethane (5.0 equiv) in methanol (4 mL/mmol of nitromethane), cooled to 0 °C, a solution of sodium hydroxide (5.0 equiv, 2.0 M in water) was added dropwise and the resulting mixture was stirred at 0 °C for 20 min. After that time, 3-chloropropiophenone was added as a solid, and 30 min later the cold bath was removed and the mixture was stirred at rt until the disappearance of the starting material on TLC (5 h). The reaction was cooled (0 °C) for quenching by addition of water and neutralization of the pH with hydrochloric acid (3 N). The aqueous layer was extracted with dichloromethane and the final organic phase was dried over magnesium sulfate, filtrated and evaporated under vacuum. The residue was purified by flash chromatography on silica gel (stepped gradient elution using mixtures petroleum ether (30/40)/ethyl acetate 8:1, 61 and 5:1) to furnish the final nitro ketone as a yellow oil in 90% yield [R_f 0.42 (silica, petroleum ether (30/40)/ethyl acetate 4:1)] δ_H (400 MHz; $CDCl_3$) 7.92 (dd, J_1 = 7.9 Hz, J_2 = 1.4 Hz, 2H, H-3, 7), 7.56 (tt, J_1 = 7.4 Hz, J_2 = 1.3 Hz, 1H, H-5), 7.45 (dd, J_1 = J_2 =

7.7 Hz, 2H, H-4, 6), 4.51 (t, J = 6.6 Hz, 2H), 3.12 (t, J = 6.8 Hz, 2H), 2.42 (quint, J = 6.7 Hz, 2H, H-9); δ_C (100 MHz; $CDCl_3$) 197.9 (C-1), 136.4 (C-2), 133.5 (CH-5), 128.7 ($2CH_{Ar}$), 127.9 ($2CH_{Ar}$), 74.7 (CH_2), 34.5 (CH_2), 21.5 (CH_2-9).

4-Nitro-1,7-diphenylheptane-1,7-dione (18) [10]

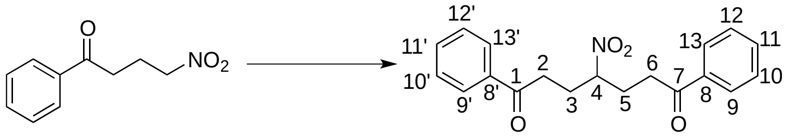

A solution of 3-chloropropiophenone (1.0 equiv) in anhydrous dichloromethane (5 mL/mmol) was prepared under nitrogen and cooled down to 0 °C. To the cold solution, triethylamine (1.0 equiv) was added and the mixture was stirred at rt until no starting material could be observed on TLC (2 h). After that time, the mixture was again taken to 0 °C and 4-nitrobutyrophenone (1.0 equiv) was added, followed by an additional 0.6 equiv of triethylamine. After stirring at 0 °C for 30 min, the mixture was warmed up to rt and stirred for 24 h. The reaction was then stopped by the addition of water, separation of the phases and extraction of the aqueous phase with dichloromethane. The organic phases were combined, dried over magnesium sulfate, filtered and evaporated under vacuum. The residue was purified by flash chromatography on silica (stepped gradient elution using mixtures petroleum ether (30/40)/diethyl ether 8:1, 4:1 and 3:1) and **18** was obtained as a white solid in 64% yield [mp 131.2–132.2 °C (petroleum ether/diethyl ether) (lit., [10], 134–135 °C); R_f 0.23 (silica, petroleum ether (30/40)/diethyl ether 3:1]; δ_H (400 MHz; $CDCl_3$) 7.93 (dd, J_1 = 7.2 Hz, J_2 = 1.3 Hz, 4H, H-9, 9', 13, 13'), 7.58 (tt, J_1 = 7.5 Hz, J_2 = 1.1 Hz, 2H, H-11, 11'), 7.46 (dd, J_1 = J_2 = 7.6 Hz, 4H, H-10, 10', 12, 12'), 4.78 (m, 1H, H-4), 3.08 (t, J = 7.5 Hz, 4H, H-2, 6), 2.39 (m, 4H, H-3, 5); MS m/z (%) 326 ($M+H^+$, 70), 279 ($M-NO_2$, 100), 278 (49).

(3*R*,5*S*,7a*r*)-3,5-Diphenylhexahydro-1*H*-pyrrolizine (17) [12]

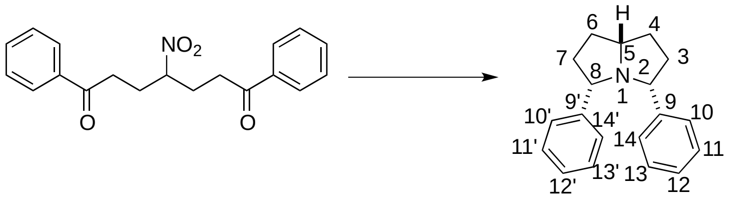

In a flask, a suspension of Raney-nickel (slurry in water) in methanol was prepared at rt and washed with methanol (×3) to partially remove the water. Solid **18** was then added and the mixture was hydrogenated at slightly higher than atmospheric pressure. After 24 h stirring at rt, no starting material could be observed on TLC and the reaction was then stopped by filtration through Celite® washing with methanol. The filtrate was evaporated under vacuum to leave a yellow oil, which was purified by flash chromatography on silica (elution with petroleum ether (30/40)/diethyl ether 25:1), to afford **17** in 30% yield [R_f 0.55 (silica, petroleum ether (30/40)/ethyl acetate 10:1)] ν_{max} (CH_2Cl_2/cm^{-1}) 2966, 2790, 1685, 1455, 754, 690; δ_H (400 MHz; $CDCl_3$) 7.00 (dd, J_1 = 7.5 Hz, J_2 = 1.6 Hz, 4H, H-10, 10', 14, 14'), 6.87 (m, 6H, H-11, 11', 12, 12', 13, 13'), 3.23 (t, J = 7.9 Hz, 2H, H-2, 8), 2.84 (septet, 1H, H-5), 2.52 (dq, J_1 = 13.3 Hz, J_2 = 8.8 Hz, 2H, H-3_A, 7_A), 2.11 (m, 2H, H-3_B, 7_B), 1.79 (m, 2H, H-4_A, 6_A), 1.70 (dq, J_1 = 10.7 Hz, J_2 = 8.4 Hz, 2H, H-4_B, 6_B); δ_C (100 MHz; $CDCl_3$) 143.5 (C-9 and 9'), 127.9 (CH-10, 10', 14 and 14'), 127.2 (CH-11, 11', 13 and 13'), 126.0 (CH-12 and 12'), 72.6 (CH-5), 65.0 (CH-2 and 8), 40.4 (CH_2-3 and 7), 26.3 (CH_2-4 and 6); HRMS (ESI$^+$) calcd for $C_{19}H_{22}N$ (M + H)$^+$ 264.1752, found 264.1760.

***tert*-Butyl 1,3-dihydroxy-2-(hydroxymethyl)propan-2-ylcarbamate** [13]

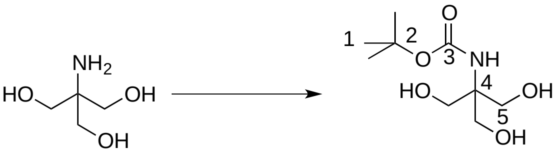

Tris(hydroxymethyl)aminomethane (3.03 g, 25 mmol) and di-*tert*-butyl dicarbonate (6.54 g, 30 mmol) were dissolved in MeOH/Et_3N (9:1) solution (75

mL). The mixture was then stirred at rt for 18 h. After this time the reaction was concentrated in vacuo and the resulting residue was redissolved in a mixture of water and EtOAc. The layers were separated and the aqueous phase was extracted with EtOAc (×10). The combined organic layers were then dried over $MgSO_4$ and the EtOAc was removed in vacuo to leave the desired product as a white solid (3.85 g, 17.4 mmol, 70%). mp 142–143 °C (literature mp 147 °C). δ_H (400 MHz, CD_3OD) 1.46 (9H, s, H1), 3.70 (6H, s, H5), 4.81 (3H, s, OH); δ_C (100 MHz, CD_3OD) 27.7 (CH_3), 60.5 (C-N), 61.9 (CH_2O), 79.6 (C-O), 157.1 (C=O). ν_{max} (neat) 3300 (O-H stretch), 1677 (C=O stretch).

***tert*-Butyl 1,3-*bis*(allyloxy)-2-(allyloxymethyl)propan-2yl carbamate (19)**
[14]

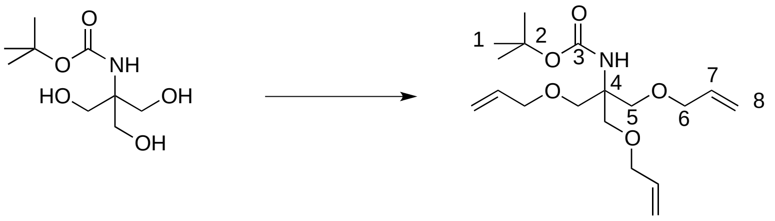

tert-Butyl 1,3-dihydroxy-2-(hydroxymethyl)propan-2-yl carbamate (3.00 g, 13.6 mmol) was dissolved in anhydrous DMF (40 mL). To the resulting solution was added allyl bromide (9.03 g, 6.46 mL, 74.6 mmol) followed by finely ground KOH (4.18 g, 74.6 mmol), which was added slowly over a period of 15 min. The mixture was then stirred at rt for 4 h. After this time the reaction was filtered and diluted with MeOH. The filtrate was concentrated in vacuo and the resulting material was then dissolved in EtOAc and washed with H_2O. The layers were separated and the aqueous phase extracted with EtOAc (×5). The combined organic layers were concentrated in vacuo to leave the crude product as a yellow oil. This material was then purified by flash chromatography on silica (10:1 pet. ether/Et_2O) to give the desired product as a colourless oil (3.08 g, 9.03 mmol, 66%). R_f 0.93 (4:1 pet. ether/EtOAc); δ_H (400 MHz, $CDCl_3$) 1.41 (s, 9H, H1), 3.68 (s, 6H, H5), 3.96 (d, J = 5.0, 6H, H6), 4.94 (s, 1H, NH), 5.14 (app. d, J = 11.0, 3H, $H8_a$), 5.23 (app. d, J = 17.0, 3H,

H8$_b$), 5.81–5.91 (m, 3H, H7); δ$_C$ (100 MHz, $CDCl_3$) 28.7 (CH_3), 58.9 (C-N), 69.5 (CH_2O), 72.6 (CH_2O), 79.2 (C-O), 117.1 (=CH_2), 135.2 (=CH), 155.2 (C=O); ν_{max} (thin film CH_2Cl_2) 3442 (N-H stretch), 1717 (C=O stretch), 1646 (C=C stretch). Spectral data is consistent with literature values [14].

(*E*)-Ethyl 4-(3-(allyloxy)-2-(allyloxymethyl)-2-(*tert*-butoxycarbonylamino)propoxy)but-2-enoate (20)

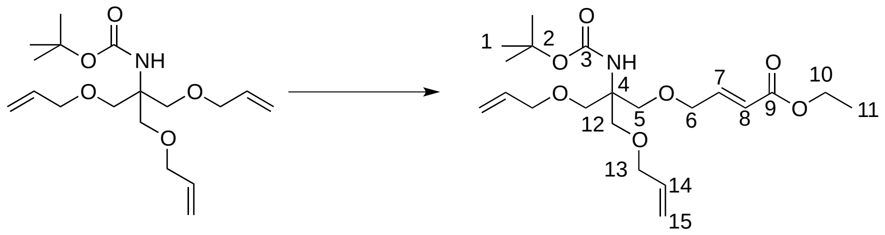

Compound **19** (68 mg, 0.2 mmol) was dissolved in ethyl acrylate (1 mL, 918 mg, 9.18 mmol). The resulting solution was degassed by bubbling with N_2 for 10 min before Grubbs II (5 mg, 0.006 mmol) was added to the reaction. The reaction was then stirred at rt under a nitrogen atmosphere for 24 h. The crude reaction mixture was then purified by flash chromatography on silica (stepped gradient; 100% pet. ether, 15:1, 10:1, 5:1 pet. ether/EtOAc) to furnish the title compound as a colourless oil (34.1 mg, 0.083 mmol, 41%). R_f 0.45 (9:1 pet. ether:EtOAc); δ$_H$ (400 MHz, $CDCl_3$) 1.22 (t, *J* = 7.0, 3H, H11), 1.35 (s, 9H, H1), 3.61 (d, *J* = 9.5, 2H, H12$_a$), 3.64 (d, *J* = 9.5, 2H, H12$_b$), 3.70 (s, 2H, H5), 3.92 (dt *J* = 5.5, 4H, H13), 4.08 (dd, *J* = 4.0, 2.0 Hz, 2H, H6), 4.13 (q, *J* = 7.0, 2H, H10), 4.87 (s, 1H, N<u>H</u>), 5.10 (app dq, *J* = 11.0, 1.0, 2H, H15$_a$), 5.19 (app dq, *J* = 17.0, 1.0, 2H, H15$_b$), 5.76–5.86 (m, 2H, H14), 5.98 (dt, *J* = 15.5, 1.0, 1H, H8), 6.85 (dt, *J* = 15.5, 3.0, 1H, H7); δ$_C$ (100 MHz, $CDCl_3$) 13.2 (CH_3), 27.4 (CH_3), 57.5 (OCH_2), 59.3 (C-N), 68.1 (OCH_2), 69.0 (OCH_2) 71.3 (2 × OCH_2), 78.1 (C-O), 115.9 (=CH_2), 120.1 (=CH), 133.7 (=CH), 143.3 (=CH), 153.8 (C=O), 165.3 (C=O). ν_{max} (thin film CH_2Cl_2) 3446 (N-H stretch), 1715 (C=O stretch),1662 (C=C stretch); HRMS (ESI) calculated for $C_{21}H_{36}NO_7$ (M + H)$^+$ 414.2492; found 414.2492.

(2*E*,2'*E*)-Diethyl 4,4'-(2-(*tert*-butoxycarbonylamino)-2-(((*E*)-4-ethoxy-4-oxobut-2-enyloxy)methyl)propane-1,3-diyl)bis(oxy)dibut-2-enoate (21)

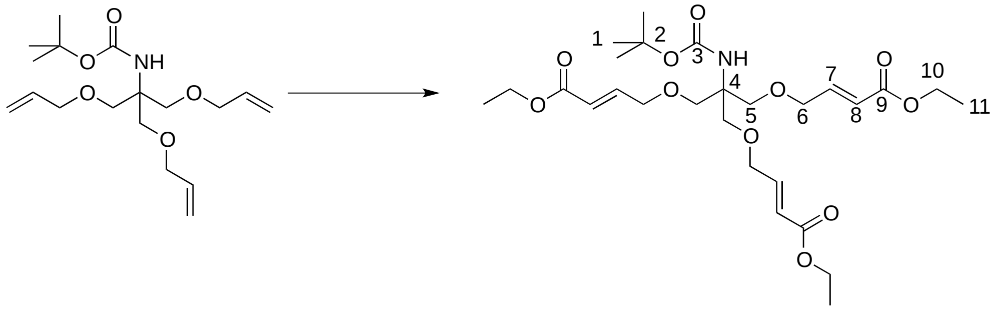

Compound **19** (239 mg, 0.7 mmol) was dissolved in ethyl acrylate (3 mL, 2.75 g, 27.5 mmol) and the resulting solution was degassed by bubbling with nitrogen for 10 min. After this time Hoveyda-Grubbs II (22 mg, 0.035 mmol) was added to the reaction and the reaction was allowed to stir at RT for 18 h. After this time the crude reaction mixture was purified by flash chromatography on silica (stepped gradient; 100% pet. ether, 15:1, 10:1, 7:1, 5:1, 2:1, 1:1, pet. ether/EtOAc). This gave the title compound as a yellow oil (283 mg, 0.51 mmol, 73%). R_f 0.26 (3:1 pet. ether/EtOAc); δ_H (400 MHz, $CDCl_3$) 1.22 (t, *J* = 7.0, 9H, H11), 1.36 (s, 9H, H1), 3.70 (s, 6H, H5), 4.08 (dd, *J* = 4.0, 2.0, 6H, H6), 4.13 (q, *J* = 7.0, 6H, H10), 4.83 (s, 1H, NH), 5.96 (dt, *J* = 15.5, 1.5, 3H, H8), 6.85 (dt, *J* = 16.0, 3.0, 3H, H7); δ_C (100 MHz, $CDCl_3$) 14.2 (CH_3), 28.3 (CH_3), 58.5 (C-N), 60.4 (OCH_2), 69.7 (OCH_2), 69.9 (OCH_2), 77.2 (C-O), 121.2 (=CH), 143.9 (=CH), 154.8 (C=O), 166.2 (C=O); ν_{max} (thin film CH_2Cl_2) 3374 (N-H stretch), 1715 (C=O stretch), 1663 (C=C stretch). HRMS (ESI) calculated for $C_{27}H_{44}NO_{11}$ (MH^+) 558.2914 found 558.2921.

Ethyl 2-(5,5-*bis*(allyloxymethyl)morpholin-3-yl)acetate (22)

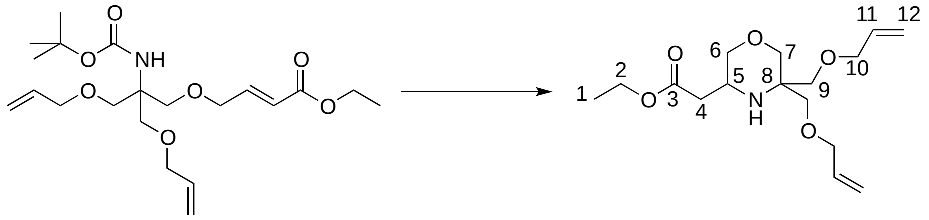

Mono-ester **21** (17.9 mg, 0.043 mmol) was dissolved in anhydrous CH_2Cl_2 (1.5 mL). $AlCl_3$ (1 M in nitrobenzene, 0.052 mL, 0.052 mmol) was added to the reaction and the mixture was stirred at rt for 20 h. The reaction was then quenched with the addition of sat. $NaHCO_3$ (aq). The resulting layers were separated and the aqueous phase extracted with EtOAc (×3). The combined organic layers were dried over $MgSO_4$ and concentrated in vacuo to leave the crude product as a solution in nitrobenzene. The crude material was then purified by flash chromatography (stepped gradient; 10:1, 5:1, 2:1 pet. ether/EtOAc) to furnish the desired product as a yellow oil (4.5 mg, 0.014 mmol, 33%); δ_H (400 MHz, $CDCl_3$) 1.18 (t, *J* = 5.0, 3H, H1), 2.17–2.18 (m, 2H, H4), 3.01–3.04 (m, 1H, $H6_a$), 3.14–3.27 (m, 3H, $H7_a$, $H9_a$, $H9_b$), 3.34–3.40 (m, 1H, H5), 3.57 (d, *J* = 9.0, 1H, $H9'_a$), 3.64 (d, *J* = 9.0, 1H, $H9'_b$), 3.63–3.72 (m, 2H, $H6_b$, $H7_b$), 3.88 (app d, *J* = 5.0, 2H, H10), 3.97–3.99 (m, 2H, H10'), 4.07 (q, *J* = 5.5, 2H, H2), 5.06–5.24 (m, 4H, H12, H12'), 5.74–5.89 (m, 2H, H11, H11'); δ_C (125 MHz, $CDCl_3$) 14.2 (CH_3), 37.6 (O=C$\underline{C}H_2$), 45.7 (NCH), 55.2 (C), 60.7 (OCH_2), 66.6 (OCH_2), 70.1 (OCH_2), 71.6 (OCH_2), 72.3 (2 × OCH_2), 72.7 (OCH_2), 116.7 (=CH_2), 134.7 (=CH), 135.0 (=C*H), 171.3 (C=O); ν_{max} (thin film CH_2Cl_2) 1732 (C=O stretch), 1642 (C=C stretch); HRMS calculated for $C_{16}H_{28}NO_5$ $(M + H)^+$ 314.1967; found 314.1954.

(2E,2'E)-Diethyl 4,4'-(5-(2-ethoxy-2-oxoethyl)morpholine-3,3-diyl)*bis*(methylene)*bis*(oxy)dibut-2-enoate (23)

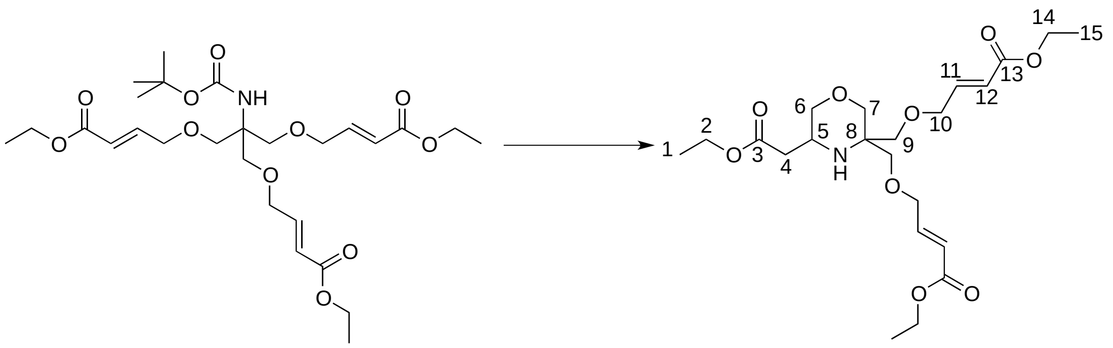

Tri-ester **21** (150 mg, 0.27 mmol) was dissolved in anhydrous CH_2Cl_2 (12 mL). To the resulting solution was added $AlCl_3$ (1 M solution in nitrobenzene, 0.54 mL, 0.54 mmol) and the reaction was then stirred at rt for 16 h, and the reaction was quenched with the addition of sat. $NaHCO_3$ (aq). The phases

were separated and the aqueous phase was extracted with CH_2Cl_2 (×3). The combined organic layers were washed with brine, dried over $MgSO_4$, and concentrated in vacuo to leave the crude product as a solution in nitrobenzene. The crude product was then purified by flash chromatography on silica (stepped gradient; 10:1, 5:1, 1:1 pet. ether/EtOAc) to furnish the title compound as a yellow oil (90.3 mg, 0.19 mmol, 73%). R_f 0.66 (EtOAc); δ_H (400 MHz, $CDCl_3$) 1.16–1.26 (m, 9H, H1, H15), 2.13 (dd, J = 16.0, 8.0, 1H, $H4_a$), 2.20 (dd, J = 16.0, 5.0, 1H, $H4_b$) 3.02 (t, J = 7.0, 1H, $H6_a$), 3.19–3.26 (m, 3H, $H7_a$, $H9_a$, $H9_b$), 3.34–3.42 (m, 1H, H5), 3.64–3.74 (m, 4H, $H6_b$, $H7_b$, $H9'_a$, $H9'_b$), 4.04–4.16 (m, 10H, H2, H10, H14), 5.94 (dt, J = 16.0, 1.5, 1H, H12), 6.01 (dt, J = 15.5, 1.5, 1H, H12'), 6.81–6.92 (m, 2H, H11, H11'); δ_C (100 MHz, $CDCl_3$) 14.6 (CH_3), 37.9 ($\underline{C}H_2C{=}O$), 46.1 (NCH), 53.8 (C), 60.7 (OCH_2), 60.8 (OCH_2), 61.1 (OCH_2), 67.9 (OCH_2), 70.4 (3 × OCH_2), 72.1 (OCH_2), 73.8 (OCH_2), 121.6 (=CH_2), 144.4 (=CH), 144.7 (=CH), 166.6 (C=O), 166.7 (C=O), 171.6 (C=O); ν_{max} (thin film, CH_2Cl_2) 1717 (C=O stretch), 1661 (C=C stretch); HRMS calculated for $C_{22}H_{36}NO_9$ $(M + H)^+$ 458.2390; found 458.2376.

References

1. Díaz-Gavilán, M.; Galloway, W. R. J. D.; O'Connell, K. M. G.; Hodgkinson, J. T.; Spring, D. R. *Chem. Comm.* **2010,** *46,* 776–778.
2. Barluenga, S.; Moulin, E.; Lopez, P.; Winssinger, N. *Chem. Eur. J.* **2005,** ***11,*** 4935.
3. Kim, S. H.; Zuercher, W. J.; Bowden, N. B.; Grubbs, R. H. *J. Org. Chem.* **1996,** ***61,*** 1073; Legeay, J. C.; Lewis, W.; Stockman, R. A. Chem. Commun. **2009,** 2207.
4. Jirgensons, A.; Kauss, V.; Kalvinsh, I.; Gold, M. R. *Synthesis* **2000,** 1709.
5. Quinet, C.; Pourdain, P.; Hermans, C.; Atesy, A.; Lucas, I.; Marko, I. E. *Tetrahedron* **2008,** *64,* 1077.
6. Basel, Y.; Hassner, A. *J. Org. Chem.* **2000,** *65*, 6368–6380.
7. Numbering pyrrolizidine: *J. Heterocyclic Chem.* **1979,** *16*, 1041; Crabb, T. A.; Newton, R. F.; Jackson, D. *Chem. Rev.* **1971,** ***71,*** 109.
8. Ayer, W. A.; Dawe, R.; Eisner, R. A.; Furuichi, K. *Can. J. Chem.* **1976,** *54,* 473; Mueller, R. H.; Thompson, M. E.; DiPardo, R. M. *J. Org. Chem.* **1984,** *49,* 2217; Gerasyuto, A. I.; Hsung, R. P. *J. Org. Chem.* **2007,** *72,* 2476.
9. The synthetic procedure is described in Scarpi, D.; Occhiato, E. G.; Guarna, A. *Tetrahedron: Asymmetry* **2005,** *16,* 1479, from 3-chloro-1-(4-chlorophenyl)propan-

1-one. Synthesis (46% yield) and characterisation of 4-nitrobutyrophenone can be found in Guarna, A.; Occhiato, E. G.; Spinetti, L. M.; Vallecchi, M. E.; Scarpi, D. *Tetrahedron* **1995,** *51,* 1775.

10. Occhiato, E. G.; Scarpi, D.; Menchi, G.; Guarna, A. *Tetrahedron Asymmetry* **1996,** *7,* 1929.

11. Smith, L. I.; Showell, J. S. *J. Org. Chem.* **1952,** *17,* 839.

12. Scarpi, D.; Occhiato, E. G.; Guarna, A. *J. Org. Chem.* **1999,** *64,* 1727.

13. Kang, J.-H.; Chung H.-E.; Kim, S.Y.; Kim, Y.; Lee, J.; Lewin, N. E.; Pearce, L. V.; Blumberg, P. M.; Marquez, V. E. *Biorg. Med. Chem.* **2003**, *11*, 2529–2539.

14. Segura, M.; Sansone, F.; Casnati, A.; Ungaro, R. *Synthesis* **2001,** 2105–2112.
