# Supplementary material for: Two-directional synthesis as a tool for diversity-oriented synthesis: Synthesis of alkaloid scaffolds
Source: Beilstein J Org Chem. 2012 Jun 6;8:850–60. doi: 10.3762/bjoc.8.95 (PMC3388873; doi:10.3762/bjoc.8.95)
Supplement: File 2 — NMR Spectra of novel compounds. [file Beilstein_J_Org_Chem-08-850-s002.pdf]

Supporting Information

for

Two-directional synthesis as a tool for diversity-oriented synthesis: Synthesis of alkaloid scaffolds

Kieron M. G. O'Connell[1], Monica Díaz-Gavilán[1,§], Warren R. J. D. Galloway[1] and David R. Spring*[1]

Address: [1]Department of Chemistry, University of Cambridge, Lensfield Rd, Cambridge, CB2 1EW, UK

[§]Present address: Departemento de Química Farmacéutica y Orgánica, Facultad de Farmacia, Campus de Cartuja, s.n. 18071 Granada, Spain

Email: David R. Spring - spring@ch.cam.ac.uk

* Corresponding author

NMR Spectra of novel compounds

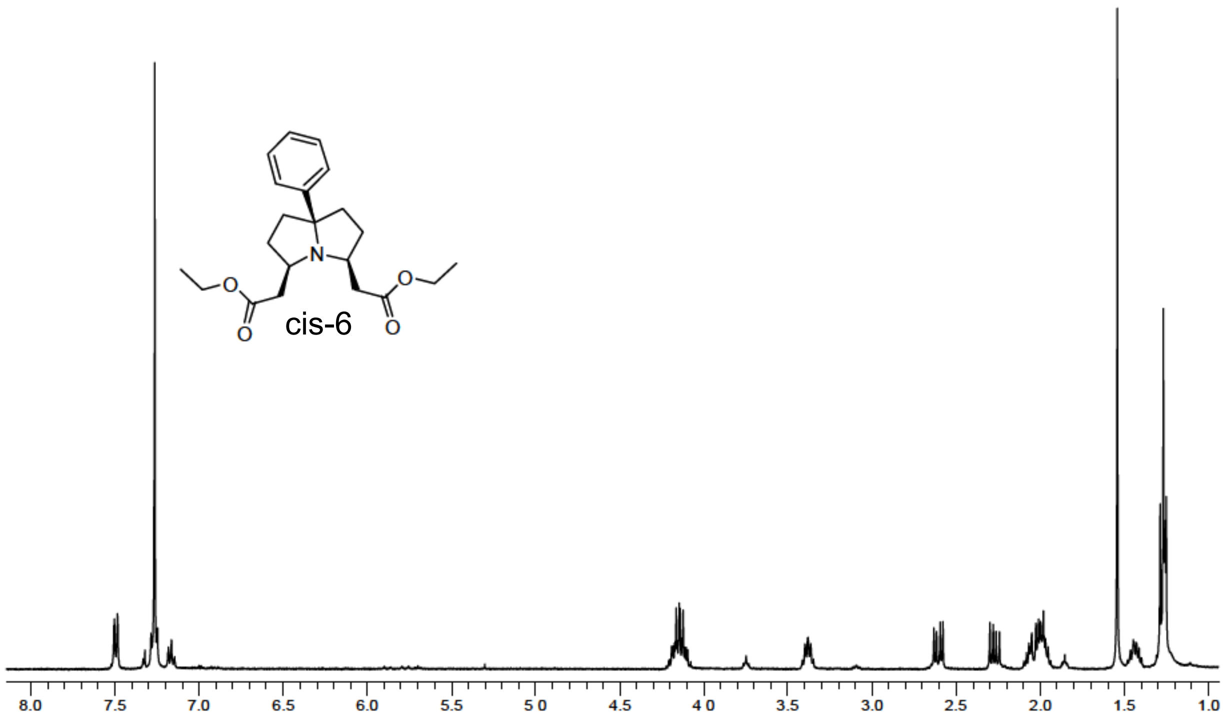
cis-6
N
O
8.0 7.5 7.0 6.5 6.0 5.5 5 0 4.5 4 0 3.5 3.0 2.5 2.0 1.5 1.0

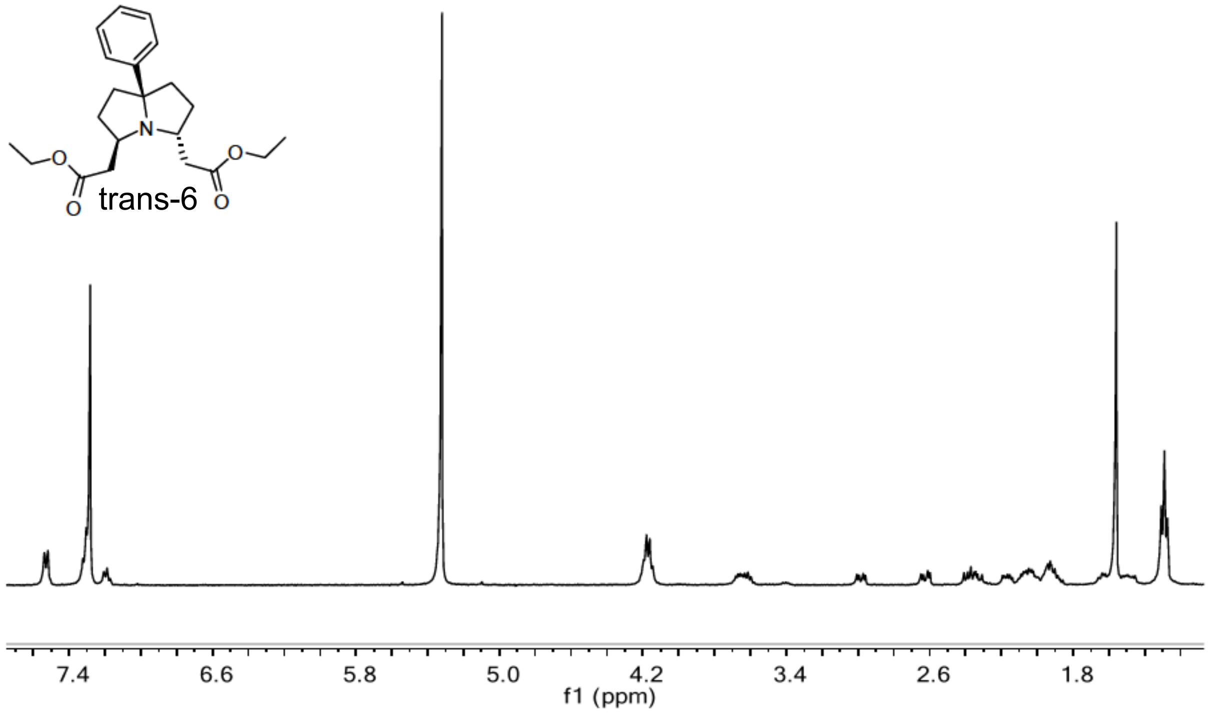
trans-6
N
O
7.4 6.6 5.8 5.0 4.2 3.4 2.6 1.8
f1 (ppm)

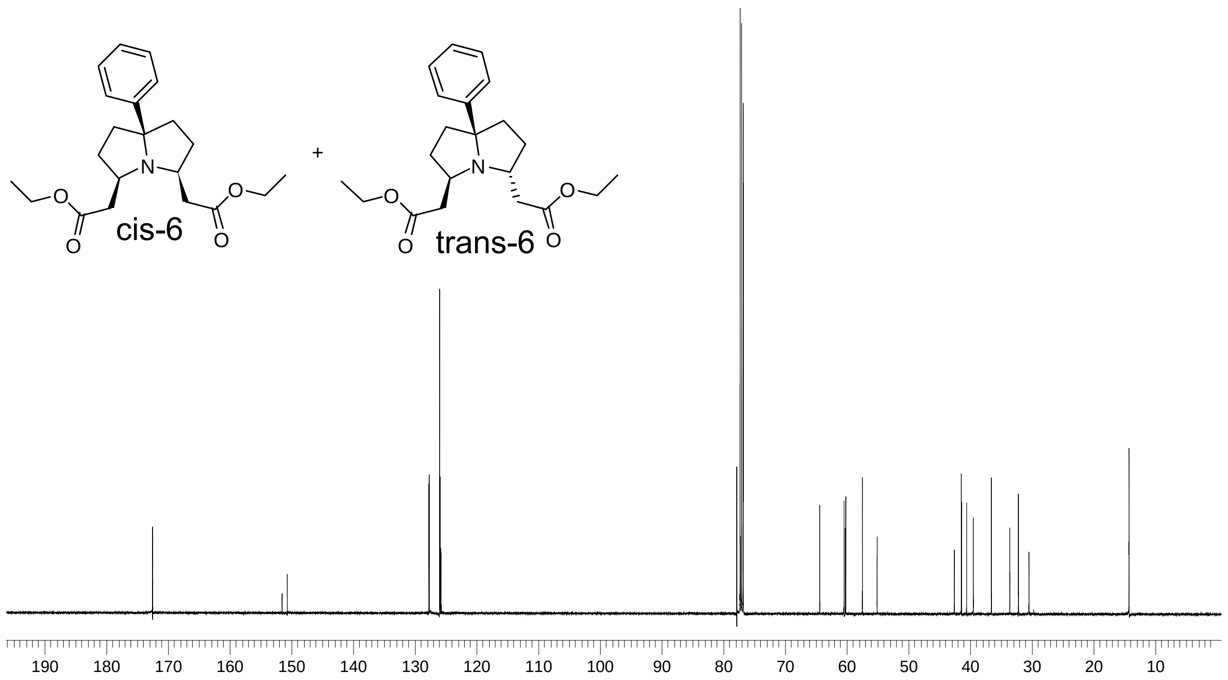
cis-6
trans-6
190 180 170 160 150 140 130 120 110 100 90 80 70 60 50 40 30 20 10

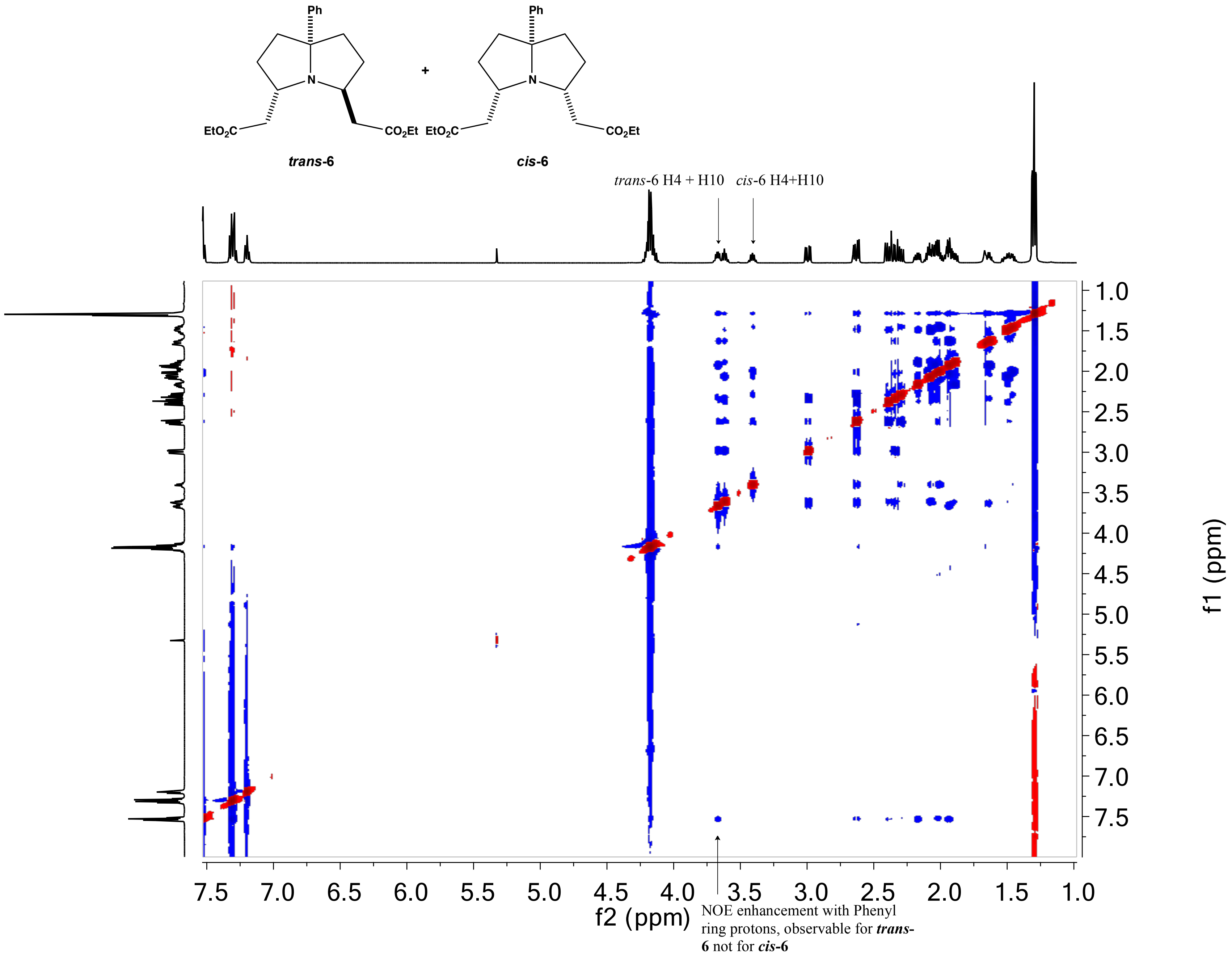
Ph
N
EtO2C
CO2Et
trans-6
+
Ph
N
EtO2C
CO2Et
cis-6
trans-6 H4 + H10
cis-6 H4+H10
f2 (ppm)
f1 (ppm)
NOE enhancement with Phenyl ring protons, observable for trans-6 not for cis-6

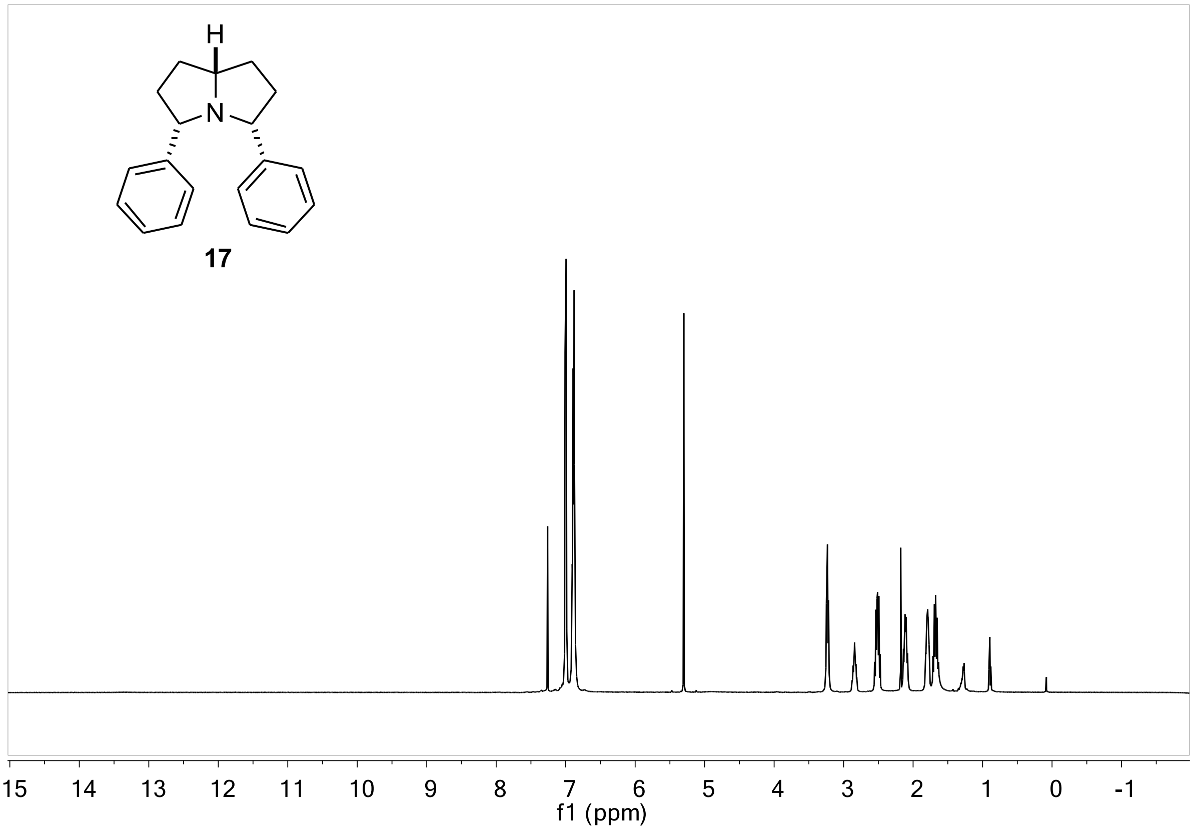
H
N
17
15 14 13 12 11 10 9 8 7 6 5 4 3 2 1 0 -1
f1 (ppm)

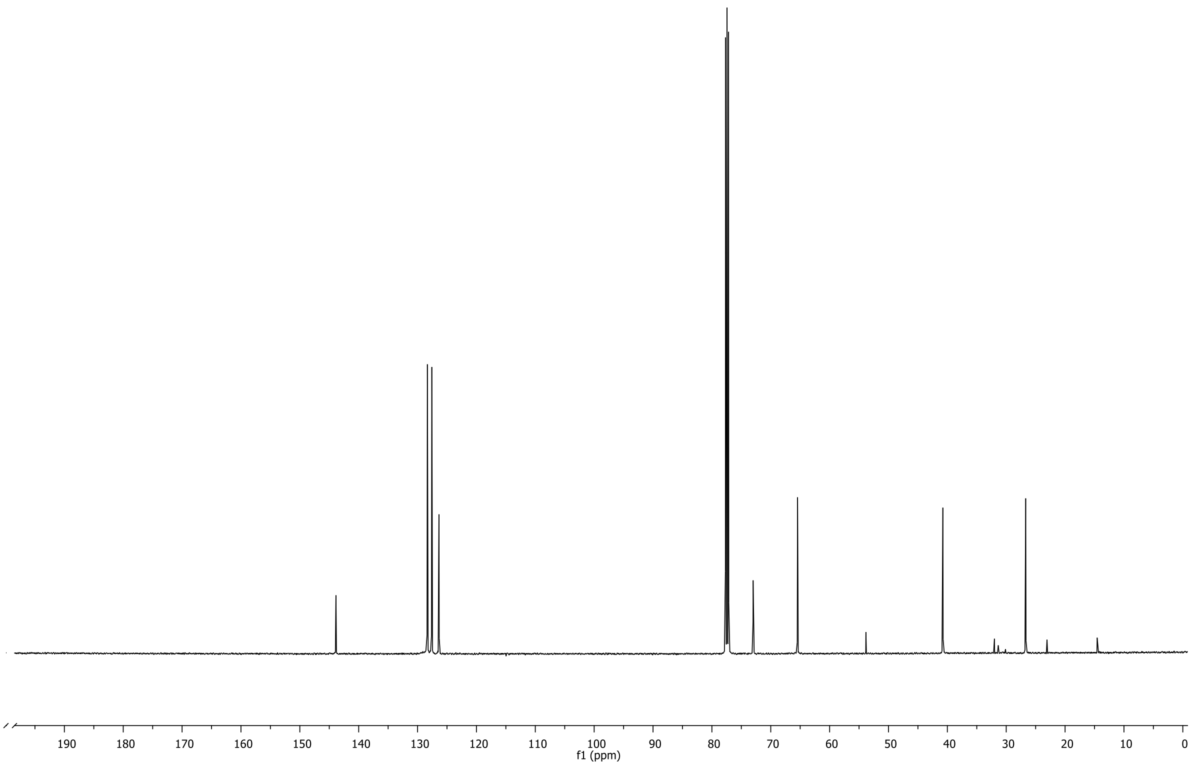
190 180 170 160 150 140 130 120 110 100 90 80 70 60 50 40 30 20 10 0
f1 (ppm)

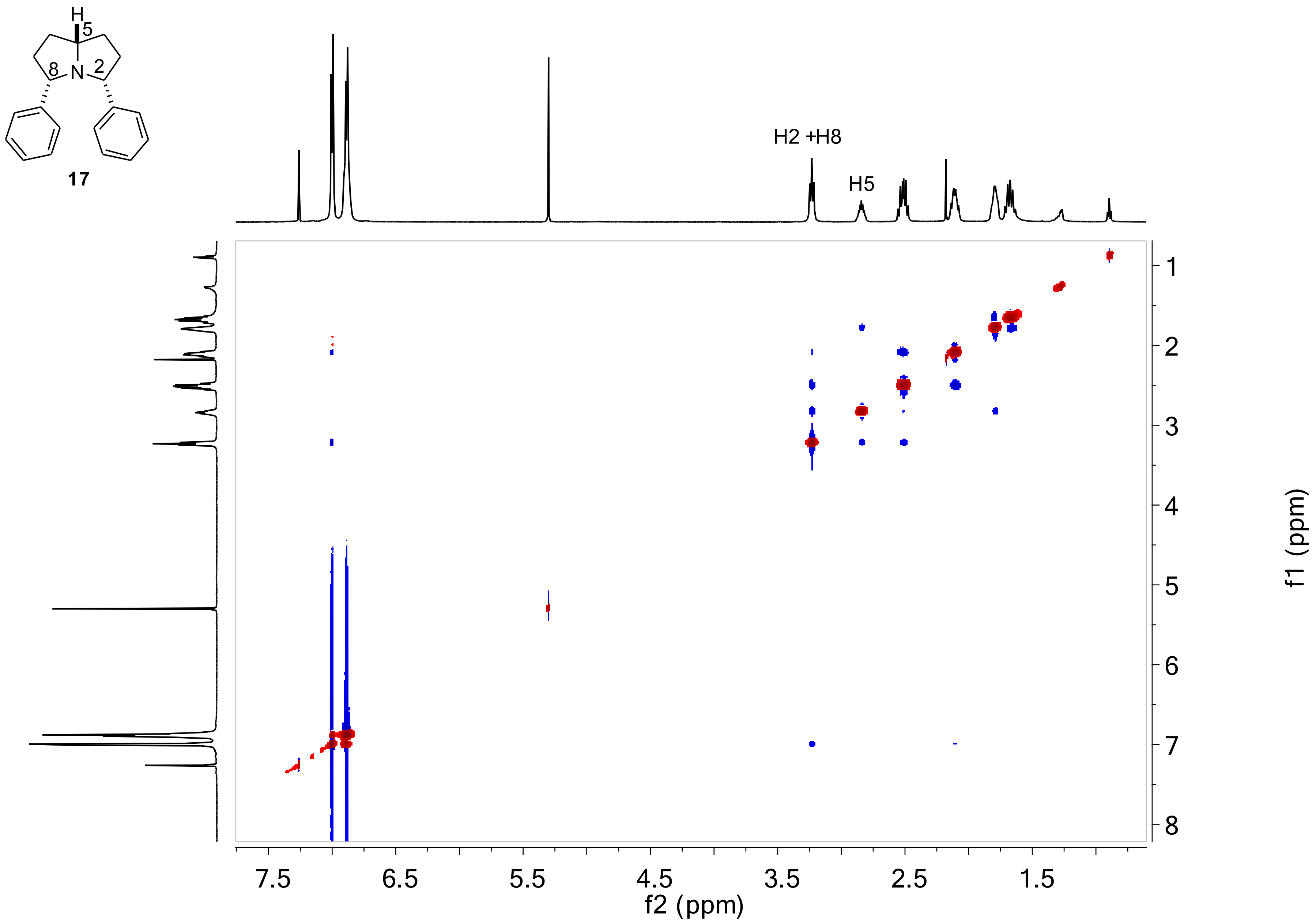

H
5
8
N
2
17
H2 +H8
H5
7.5
6.5
5.5
4.5
3.5
2.5
1.5
f2 (ppm)
1
2
3
4
5
6
7
8
f1 (ppm)
